# Shared Multidrug Resistance Patterns in Chicken-Associated *Escherichia coli* Identified by Association Rule Mining

**DOI:** 10.3389/fmicb.2019.00687

**Published:** 2019-04-12

**Authors:** Casey L. Cazer, Mohammad A. Al-Mamun, Karun Kaniyamattam, William J. Love, James G. Booth, Cristina Lanzas, Yrjö T. Gröhn

**Affiliations:** ^1^Department of Population Medicine and Diagnostic Sciences, Cornell University College of Veterinary Medicine, Ithaca, NY, United States; ^2^Department of Epidemiology of Microbial Diseases, Yale University School of Public Health, New Haven, CT, United States; ^3^Department of Population Health and Pathobiology, North Carolina State University College of Veterinary Medicine, Raleigh, NC, United States; ^4^Department of Biological Statistics and Computational Biology, Cornell University College of Agriculture and Life Sciences, Ithaca, NY, United States

**Keywords:** association rule mining, antimicrobial resistance, *Escherichia coli*, machine learning, multidrug resistance, foodborne bacteria

## Abstract

Using multiple antimicrobials in food animals may incubate genetically-linked multidrug-resistance (MDR) in enteric bacteria, which can contaminate meat at slaughter. The U.S. National Antimicrobial Resistance Monitoring System tested 14,418 chicken-associated *Escherichia coli* between 2004 and 2012 for resistance to 15 antimicrobials, resulting in >32,000 possible MDR patterns. We analyzed MDR patterns in this dataset with association rule mining, also called market-basket analysis. The association rules were pruned with four quality measures resulting in a <1% false-discovery rate. MDR rules were more stable across consecutive years than between slaughter and retail. Rules were decomposed into networks with antimicrobials as nodes and rules as edges. A strong subnetwork of beta-lactam resistance existed in each year and the beta-lactam resistances also had strong associations with sulfisoxazole, gentamicin, streptomycin and tetracycline resistances. The association rules concur with previously identified *E. coli* resistance patterns but provide significant flexibility for studying MDR in large datasets.

## Introduction

Although bacteria had antimicrobial resistance genes prior to the discovery and clinical use of antimicrobials in the 1940s, antimicrobial use (AMU) selects for antimicrobial resistance (AMR) in both pathogenic and non-pathogenic bacteria (Knapp et al., 2010). Increased AMR can emerge and persist in food-borne pathogens through the use of antimicrobials in food-producing animals (Marshall and Levy, 2011). AMU in food-producing animals is projected to increase by 67% from 2010 to 2030 due to increasing demands for meat products and human population growth (Van Boeckel et al., 2015). Each instance of AMU selects for AMR directly by favoring the growth or persistence of phenotypically resistant bacteria in treated individuals (Lipsitch and Samore, 2002). AMU also indirectly selects for AMR by increasing the prevalence of resistant phenotypes in a population, thereby increasing the risk of future resistant infections (Lipsitch and Samore, 2002). Resistant pathogens can spread from colonized food-producing animals to a limited number of humans through direct contact and to broader human populations via the food chain if meat is contaminated with pathogens at slaughter (Marshall and Levy, 2011; Klous et al., 2016).

Pathogens with multi-drug resistance (MDR) are a larger public health threat than pathogens with one drug or class resistance because it can be difficult or impossible to find an antimicrobial effective against MDR infections. MDR is not just a consequence of individual drug resistances occurring together by chance. Chang et al. (2015) found that MDR often occurs at higher rates than expected by chance and describe several mechanisms that contribute to the emergence of MDR. Some biological mechanisms or alterations (e.g., efflux pumps) protect against several antimicrobial drugs or classes, creating cross-resistance. Additionally, resistance genes can be genetically linked by occurring on the same mobile element or chromosome region and therefore tend to be inherited or transferred together (co-resistance) (Chang et al., 2015). MDR occurs naturally in bacteria that have never been exposed to anthropogenic antimicrobials (Brown and Balkwill, 2009), however AMU can select for MDR in two key ways. Changes in AMU or the use of multiple antimicrobials together can lead to MDR emergence through genetic capitalism, whereby bacteria that are already resistant to one antimicrobial have a fitness advantage and are therefore more likely to acquire additional resistances via mutation, recombination or horizontal gene transfer (Chang et al., 2015). Knowledge of common MDR patterns in specific bacteria-host-environment situations can help guide antimicrobial therapy and stewardship (Pitout and Laupland, 2008).

The National Antimicrobial Resistance Monitoring System (NARMS) was established in 1996 as a national surveillance system in the United States to monitor AMR in zoonotic, food-borne bacteria (including *Salmonella enterica, Campylobacter* species, and *Escherichia coli)*. It includes samples from clinically ill humans, hazard-based samples of food-animal carcasses at slaughter plants, and systematic samples of retail meats (Karp et al., 2017). NARMS began monitoring *E. coli* isolated from chicken carcasses in 2000 and from retail chicken in 2002 as an indicator organism for AMR in gram negative bacteria (Karp et al., 2017). NARMS reports the prevalences and trends of individual drug resistances, MDR patterns, and MDR prevalence but also makes the isolate-level minimum inhibitory concentration (MIC) data available to the public (Food and Drug Administration, 2016). Biologic and genetic linkages between resistances can be inferred from this isolate-level data. In general, AMR data can be analyzed as continuous (disk diffusion zone diameters), ordinal (MIC) or dichotomous (susceptible or resistant) data. However, it is challenging to analyze associations within AMR data because the data often violate assumptions for classical statistical tests and models. For example, MIC and disk diffusion distributions tend to be non-Gaussian (Wagner et al., 2003) and dichotomous interpretations tend to be sparse and over dispersed (Zawack et al., 2016, 2018). Several techniques and statistical models have been applied to NARMS data and similar AMR datasets in order to understand relationships between drug resistances. Graphical Markov networks have revealed pairwise associations between drug resistances (Love et al., 2016, 2018). Log-linear models have been used to understand higher-order associations between drug resistances but these models violate key assumptions when there are fewer than 5 isolates expected in each combination of resistances or susceptibilities (i.e., sparsity) (Zawack et al., 2018). Finally, Bayesian approaches can estimate interactions between drug resistances (Ludwig et al., 2013; Zawack et al., 2018), and have identified three-way and four-way interactions between resistances in NARMS data (Zawack et al., 2018).

Association rule mining is an unsupervised machine learning method that is commonly used to identify patterns in customer purchasing at retail stores in order to improve marketing and to make better business decisions (Tan et al., 2018). Rule mining can efficiently discover associations between binary or categorical variables in large, sparse datasets (Agrawal et al., 1993; Tan et al., 2018). Although the definition of “large dataset” or “big data” varies, the Apriori algorithm commonly used for association rule mining was developed for datasets with tens of thousands of retail transactions (Agrawal et al., 1993). Sparse data refers to situations where very few observations have non-zero values. When AMR data is dichotomized into susceptible (e.g., “0”) and resistant (e.g., “1”) interpretations, the data may be sparse when the prevalence of resistance is low. Therefore, association rule mining is well-suited to analyzing AMR surveillance data, which often contains thousands of isolates with low resistance prevalence for some drugs (Love et al., 2016; MacKinnon, 2017). In addition, association rule mining does not make assumptions of Gaussian data or expected count values. It has been suggested that association rule mining is more sensitive than regression techniques or chi-square tests for finding relationships between exposures and disease outcomes in clinical data (Cleophas and Zwinderman, 2015). Association rules have been used in studies of nosocomial infection trends (Ma et al., 2003; Tsymbal, 2005; Giannopoulou et al., 2007), antibiogram analyses (Ma et al., 2003; Tsymbal, 2005; Gerontini et al., 2011), and susceptibility testing validation (Lamma et al., 2001). Since antimicrobial susceptibility results can be interpreted as binary variables (e.g., susceptible or resistant to a given antimicrobial), association rules can identify multi-way relationships between individual antimicrobial susceptibilities that result from cross-resistance or co-resistance.

The objective of the current study is to uncover significant MDR patterns in antimicrobial susceptibility testing data with association rule mining. Multi-drug resistance commonly refers to resistance to three or more drug classes (Magiorakos et al., 2012); we refer to MDR as resistance to two or more drug classes (Table 1) since association rules involve two or more antimicrobials. MDR is difficult to study with regression model techniques because there are many potential outcomes (i.e., MDR patterns). However, association rule mining efficiently identifies the strongest patterns in a dataset and analysis of the association rules can provide insight into the strength of relationships between individual antimicrobial susceptibilities. We apply association rule mining to NARMS resistance data of *Escherichia coli* isolated from chicken carcasses and retail meat in order to identify important associations between two or more antimicrobial drugs and investigate temporal trends and differences between slaughter and retail MDR patterns.

**Table 1:** Antimicrobial classes and resistance breakpoints of drugs used between 2004 and 2012 in the antimicrobial susceptibility testing of *Escherichia coli* isolated from chicken carcasses and chicken retail meat by the National Antimicrobial Resistance Monitoring System.

**Class**	**Drug**	**Abbreviation**	**Years Tested**	**Resistance Breakpoint**
β-lactams	Ampicillin	AMP	2004–2012	≥32
	Amoxicillin–Clavulanic Acid	AMC	2004–2012	≥32
	Ceftriaxone	AXO	2004–2012	≥4
	Cefoxitin	FOX	2004–2012	≥32
	Ceftiofur	TIO	2004–2012	≥8
Aminoglycosides	Amikacin	AMI	2004–2010	≥64
	Gentamicin	GEN	2004–2012	≥16
	Kanamycin	KAN	2004–2012	≥64
	Streptomycin	STR	2004–2012	≥64
Sulfonamides	Trimethoprim–Sulfamethoxazole	COT	2004–2012	≥4
	Sulfisoxazole	FIS	2004–2012^*^	≥512
Quinolones	Ciprofloxacin	CIP	2004–2012	≥1
	Nalidixic Acid	NAL	2004–2012	≥32
Tetracyclines	Tetracycline	TET	2004–2012	≥16
Phenicols	Chloramphenicol	CHL	2004–2012	≥32
Macrolides	Azithromycin	AZI	2011–2012	≥32

* *Except 2007 slaughter isolates*.

## Methods

### Data Sources

Antimicrobial susceptibility testing data from *Escherichia coli* isolated from chicken carcasses since 2000 and from retail chicken meat since 2002 as part of NARMS surveillance is publicly available (Food and Drug Administration, 2016). Data from 2004 to 2012 (14,418 isolates) were used for this study because of changes in NARMS sampling strategies (Karp et al., 2017) and for consistency with previous studies of AMR associations in NARMS isolates (Love et al., 2016). Each isolate was tested against 12 to 25 antimicrobial drugs using the Sensititre system (National Antimicrobial Resistance Monitoring System, 2016a). The MIC results of the 15 most commonly tested antimicrobials plus azithromycin were used for this study (Table 1). Each isolate was classified as resistant or susceptible based on published MIC breakpoints (Love et al., 2016; National Antimicrobial Resistance Monitoring System, 2017). Resistance data were separated by year and source (slaughter and retail) into 18 datasets for association rule mining. The prevalence of resistance against the 16 included antimicrobials in each year-source dataset is given in Table 2.

**Table 2:** Number (N) of *Escherichia coli* isolates from chicken carcasses (slaughter) and chicken meat (retail) between 2004 and 2012 and the prevalence of resistance to 16 antimicrobials.

			Prevalence of resistance (%)^*^															
**Source**	**Year**	**N**	**AMC**	**AMP**	**AXO**	**FOX**	**TIO**	**AMI**	**GEN**	**KAN**	**STR**	**COT**	**FIS**	**CIP**	**NAL**	**TET**	**AZI**	**CHL**
Slaughter	2004	1697	8.8	17.6	7.2	8.2	4.9	0	39.1	11.5	64.1	10.7	53.2	0.2	6.8	50.3	NT	1
	2005	2232	10.6	22	9	9.9	6.5	0	36.7	10.3	58.1	10.4	51.9	0.5	7.5	48.9	NT	1
	2006	1357	16	25.6	14.7	15	10.2	0	33.1	9.1	49.5	8.4	48.6	0.1	5.4	49	NT	1.9
	2007	1510	11.2	18.7	10.3	10.3	7	0	38	7.7	47	7.9	NT	0.1	4.2	40.2	NT	2.3
	2008	986	13.7	23.5	13.5	13.8	10.4	0	44.5	10.2	54.6	9.1	52.7	0.6	6	47.4	NT	1
	2009	876	12.4	19.9	11.5	11.4	9.5	0	43.4	7.9	49.9	7	52.6	0.5	3.2	49.2	NT	1.1
	2010	941	12.4	22.2	12.3	12.5	10	0	43	6.4	49.1	6.3	51.8	0.2	3.4	42.8	NT	0.7
	2011	614	9.4	16	9.3	9.1	6.8	NT	42.8	5.7	50.8	4.2	54.7	0.3	2.3	46.6	0.2	2.1
	2012	990	9	17.7	8.8	9.2	7.6	NT	42.1	7.3	42.8	6.5	47.6	0.5	2.4	45.4	0.5	1.9
Retail	2004	400	10	17	6.5	8.2	5.8	0	30	6.8	56.8	4.2	41.2	0	7	48	NT	1.8
	2005	393	12.2	24.7	10.2	11.2	8.7	0	37.7	7.1	50.6	7.4	48.1	0	6.6	46.6	NT	0.5
	2006	418	11.5	20.1	9.1	11.2	8.6	0	37.3	11.5	48.1	8.9	46.9	0	5	50.5	NT	2.6
	2007	299	7.4	18.1	6.4	7.4	6	0	34.4	9	46.8	5	42.1	0	3	40.5	NT	2
	2008	306	11.8	23.5	11.1	11.8	10.8	0	34	6.9	43.8	3.6	39.2	0	2.9	43.8	NT	1
	2009	315	13.3	22.3	12.4	13.3	11.7	0	34.3	5.4	38.1	2.2	40.6	0.3	2.9	41.6	NT	0.6
	2010	357	6.7	16.5	6.4	6.7	5.6	0	31.9	6.2	39.2	4.2	38.9	0.6	3.6	38.9	NT	1.4
	2011	341	14.1	26.4	12.6	13.2	12.3	NT	38.4	5.6	43.4	2.3	44.3	0	2.3	40.8	0	1.2
	2012	386	7.8	15.8	7.8	7.8	7.5	NT	30.6	5.7	39.6	2.6	37.8	0	1.8	39.4	0	0.3

*Drug name abbreviations are given in Table 1*.

* *NT indicates that the antimicrobial was not tested in that year and/or source*.

### Association Rule Mining

#### Background

Association rule mining is an unsupervised machine learning technique for identifying patterns and relationships in large, binary datasets (Tan et al., 2018). Rule mining terminology reflects its classical application to market basket data (i.e., purchase records). The binary data is arranged as *transactions* and *items*, with one transaction in each row and one item in each column. An example antimicrobial susceptibility dataset is given in Table 3; each isolate is considered a transaction and each antimicrobial is an item. Resistance to an antimicrobial is recorded as “1” and susceptibility as “0.”

**Table 3:** Example binary antimicrobial susceptibility testing dataset for six isolates (*transactions*) and five antimicrobials (*items*) with resistance indicated as 1 and susceptibility as 0.

**Isolate ID**	**Antimicrobial**				
	**A**	**B**	**C**	**D**	**E**
1	1	1	1	0	1
2	1	0	1	1	1
3	0	1	1	0	1
4	1	0	1	1	1
5	0	0	1	1	1
6	1	0	0	1	1
*Support count*	4	2	5	4	6
*Support*	0.67	0.33	0.83	0.67	1

*The support count and support (prevalence of resistance) for each individual antimicrobial is also given. Itemsets are combinations of items present in the transactions. For example, the itemset [A,B,C] is in transaction 1*.

An *itemset* is a combination of zero or more items (e.g., antimicrobial resistances). A transaction contains an itemset if all the items in the itemset appear in the transaction. For example, in Table 3 the itemset [B] is contained in isolates 1 and 3; the itemset [A, D] is contained in isolates 2, 4 and 6; and the itemset [A, B, C] is contained in isolate 1. The number of possible itemsets, excluding the null set of zero items, is 2^*k*^ − 1, where *k* is the number of items in a dataset. The example dataset in Table 3 has five items (antimicrobials) and 31 potential itemsets. The NARMS datasets used in this study include at most 15 antimicrobials and 32,767 potential combinations of resistances.

The *support count* of an itemset is the number of transactions that contain that itemset and the *support* of an itemset is the proportion of transactions that contain that itemset. For example, itemset [B] is contained in two out of six isolates in Table 3 so its support count is two and its support is 0.33; itemset [A,D] has a support count of three and a support of 0.5. A *frequent itemset* is an itemset with a support greater than or equal to a user-defined minimum support (*minsup*). If the minimum support for the example in Table 3 is 0.4, then [B] is an *infrequent itemset* and [A,D] is a frequent itemset. The support (i.e., prevalence of resistance) for each single-antimicrobial itemset (e.g., [AMP]) in the NARMS datasets is given in Table 2.

*Rules* are expressed as X → Y, where X and Y are disjoint itemsets containing one or more items. Y is referred to as the consequent or right-hand side; X is referred to as the antecedent or the left-hand side. In classical market basket analysis, this rule implies that customers who purchase all the items in X also buy all the items in Y. With respect to antimicrobial resistance, this rule implies that isolates resistant to the antimicrobials in X are also resistant to the antimicrobials in Y. A dataset can contain 3^*k*^ − 2^*k*+1^ + 1potential rules; the example dataset in Table 3 has 180 possible rules and the NARMS datasets in Table 2 have at most 14,283,372 rules when 15 antimicrobials are tested. Note that there are more potential rules than potential itemsets and that both grow exponentially with the number of items in the dataset. Rules can be described by many quality measures (Hahsler, 2015; Hahsler et al., 2018) but are commonly evaluated with support and *confidence*, which is the conditional probability of the consequent given the antecedent. Whereas, support is a symmetric quality measure (the support of X → Y is the same as Y → X), confidence is asymmetric. In Table 3, the rule A → C has a confidence of 0.75 but the rule C → A has a confidence of 0.6. The user-defined minimum confidence (*minconf* ) is used to select reliable rules from all possible rules.

#### Frequent Itemset and Rule Generation

**Figure 1:**
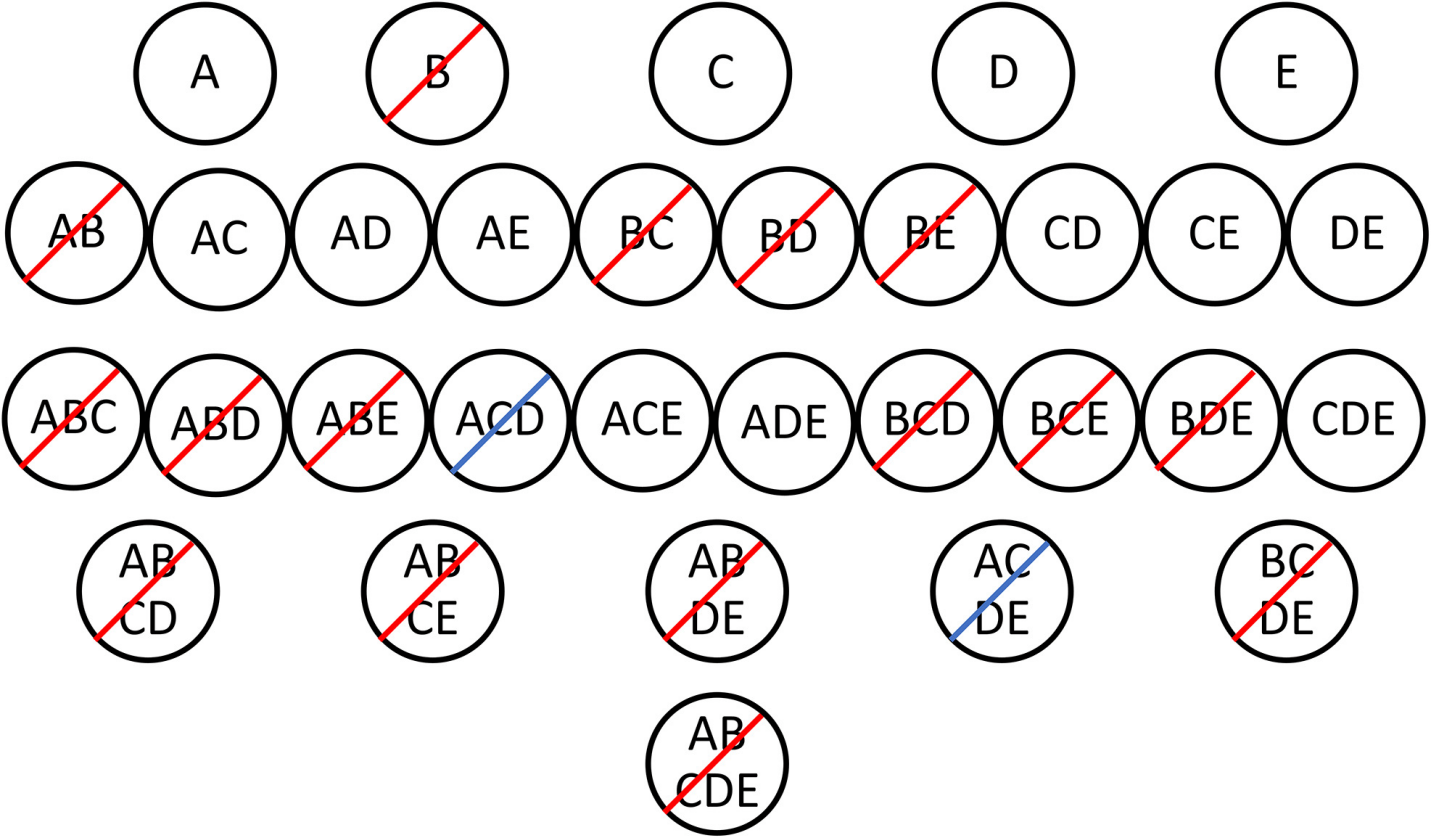
Frequent itemset generation from the Table 3 dataset using the Apriori algorithm. Each candidate itemset is represented by a black circle. The itemsets with a red slash are eliminated after examining the top row of itemsets and comparing the support of each itemset to the minimum support of 0.4. [B] has a support of 0.33 and therefore all of its supersets must have a support less than or equal to 0.33. All remaining itemsets in the second row are frequent with a support >0.4. Itemsets with a blue slash are eliminated after calculating the support for three-item itemsets.

The discovery of association rules requires, first, frequent itemset generation and, second, rule generation. The Apriori algorithm (Agrawal et al., 1993; Hahsler et al., 2018) efficiently finds frequent itemsets by pruning candidate itemsets based on the minimum support. Since the support of an itemset must be less than or equal to the support of its subsets, the algorithm looks first at the smallest itemsets (with one item) and eliminates any that do not meet the minimum support requirement. Subsequently, all candidate itemsets that include an infrequent item can be eliminated because they will not meet the minimum support. This is illustrated in Figure 1 using data from Table 3. If the minimum support is 0.4, then [B] is an infrequent itemset because its support is 0.33. The algorithm then generates possible two-item itemsets and eliminates itemsets that include B because they must also have a support less than or equal to 0.33. The supports of the remaining two-item itemsets are calculated and compared to the minimum support. This process continues until all itemsets of a given size are determined to be infrequent or the algorithm reaches the largest candidate itemset. This method efficiently identifies the frequent itemsets without having to calculate the support of each possible itemset (Agrawal et al., 1993). In Figure 1, 16 of the 31 possible frequent itemsets have been eliminated after examining the support of just five one-item itemsets.

Rules are generated from the frequent itemsets, which guarantees that the support of the rule is greater than or equal to the minimum support. Frequent itemsets are partitioned into two non-overlapping subsets, the antecedent and consequent. Although the antecedent and consequent can both contain more than one item (Tan et al., 2018), the “arules” package in R (version 3.4.3, R Core Team, 2017) restricts the consequent to just one item (Hahsler et al., 2009). For example, itemset [A, C, E] can be partitioned into rules [A, E] → C, [A, C] → E, and [C, E] → A. Rules with a confidence smaller than the minimum confidence are eliminated.

#### Implementation

Association rule mining of the NARMS data was implemented in R using the package “arules” (Hahsler et al., 2018). The minimum support and minimum confidence were both set to 1/(Number of Isolates)in order to avoid excluding antimicrobials with rare resistance (i.e., CIP). Rules including at least two antimicrobials were extracted from each of the 18 year-source datasets. Forty-six quality measures are available in “arules” (Hahsler et al., 2018) for evaluating rules (Table S1). Many quality measures are correlated and will produce similar rankings of rules (Tan et al., 2018). Therefore, principal component analysis (PCA) was used to select a small number of quality measures that capture the most variation in rule quality across the 46 quality measures evaluated (Martínez-Ballesteros et al., 2014).

**Figure 2:**
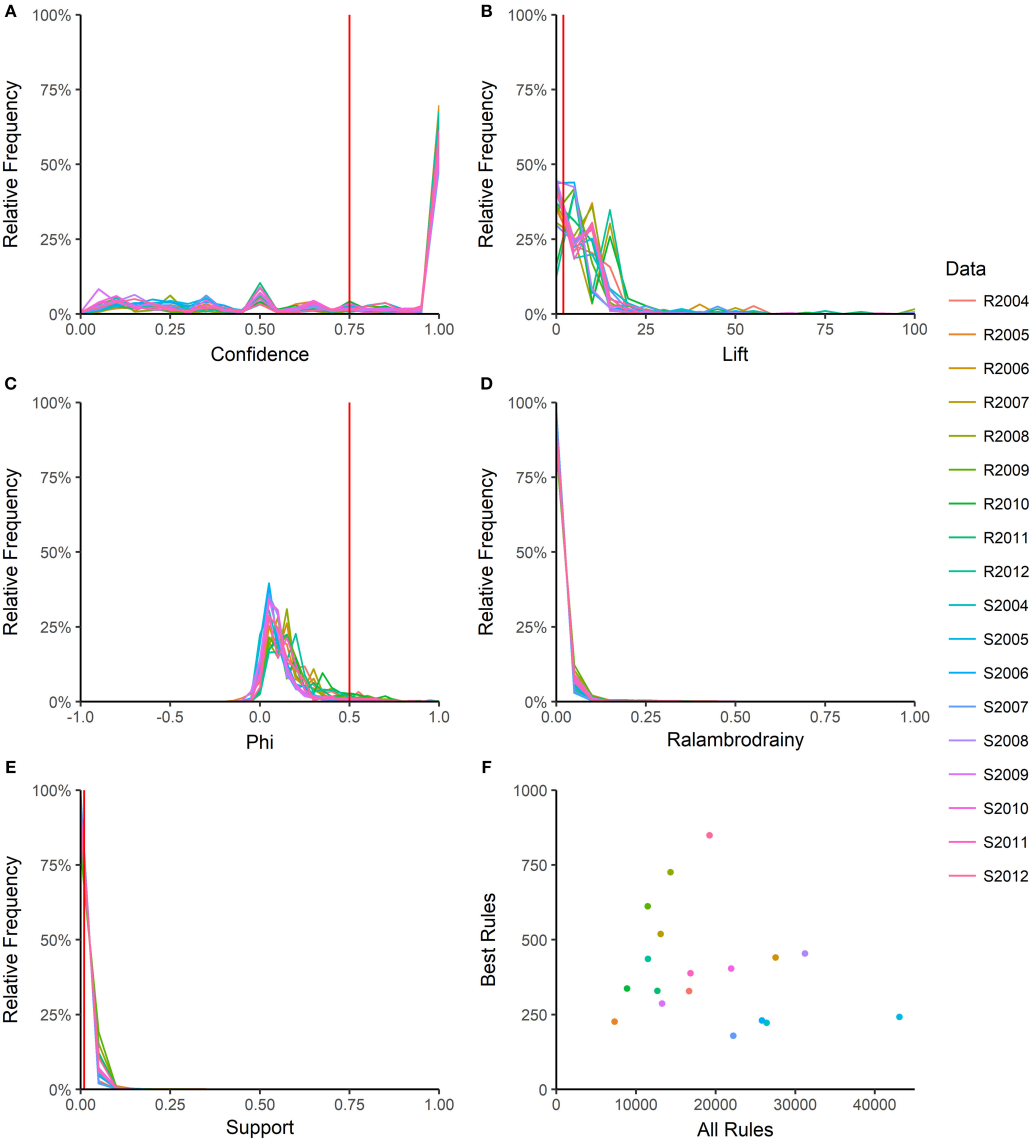
Number of association rules and distribution of rule quality measures. Five rule quality measures were calculated: confidence **(A)**, lift **(B)**, phi **(C)**, ralambrodrainy **(D)**, and support **(E)**. Frequency polygons for each year-source dataset are shown R: retail; S: slaughter. The number of rules before (all rules) and after (best rules) pruning with confidence >0.75, support >0.01, lift >2, and absolute value of phi >0.5 are shown in **(F)**. Ralambrodrainy was not used for pruning rules because a very small cut-off (>0.005) resulted in too few rules for analysis.

For each rule in the 18 year-source datasets, the 46 quality measures were calculated; rules were deleted listwise if a measure could not be calculated or had an infinite value. The function “prcomp” in R (package “stats”) was used to calculate the first four principal components (PC) in each dataset and the five quality measures with the greatest loadings in each PC were identified (Table S2). One quality measure with a high loading in all or almost all datasets was selected from each PC, resulting in four measures (confidence, lift, phi, ralambrodrainy) that captured a large proportion of the information contained in all the rule quality measures (Martínez-Ballesteros et al., 2014). The distributions of these four quality measures within each year-source dataset were examined (Figure 2) and cut-off values for confidence, lift, phi and support were selected to prune the number of rules in each dataset to 1,000 or fewer. Ralambrodrainy measure was not used for pruning because the distribution was narrow (Figure 2) and even a very small cut-off (0.005) resulted in fewer than 100 rules in many datasets. The pruned rule-sets are referred to as the best-rules and were used to compare patterns of antimicrobial resistance across years and sources.

### Comparison of Rule-Sets

The best-rules from each year-source dataset were compared with graphical and numerical methods. Temporal trends in MDR patterns were analyzed with the rule overlap ratio and cumulative rule stability. The rule overlap ratio (Dudek, 2010) gives the proportion of rules shared between two rule-sets out of all rules present in the rule-sets. Cumulative rule stability (CRS) (Dudek, 2010) averages the rule overlap over time for consecutive rule-sets. Rules that are shared between two rule-sets are compared by averaging the absolute difference in support or confidence of the shared rules (modified support difference, modified confidence difference) (Dudek, 2010).

(1) $$rule\_overlap\left(R_{1},R_{2}\right)=\frac{\left|R_{1}\cap R_{2}\right|}{\left|R_{1}\cup R_{2}\right|}$$

(2) $$CRS_{i}=\{\begin{array}{c} rule\_overlap\left(R_{i},R_{i+1}\right),\text{ if }i=1\\ \frac{1}{i}\left[\left(i-1\right)CRS_{i-1}+rule\_overlap\left(R_{i},R_{i+1}\right)\right],\text{ if }i>1\\ i=\text{year index} \end{array}$$

(3) $$sup\_diff=\frac{\sum_{r\in R_{1}\cap R_{2}}\left|sup\left(r,D_{1}\right)-sup\left(r,D_{2}\right)\right|}{\left|R_{1}\cap R_{2}\right|}$$

(4) $$conf\_diff=\frac{\sum_{r\in R_{1}\cap R_{2}}\left|conf\left(r,D_{1}\right)-conf\left(r,D_{2}\right)\right|}{\left|R_{1}\cap R_{2}\right|}$$

Rules were decomposed into antimicrobial nodes and undirected edges for graphical visualization with Gephi (Bastian et al., 2009). For example, the rule [A, E] → C decomposes into nodes (A, E, C) and undirected edges (A – C, E – C). Redundant edges were removed, and edges were weighted by the number of times each edge appeared in the best-ruleset. The nodes (antimicrobials) were assigned groups based on antimicrobial classes (Table 1). The modularity (Newman and Girvan, 2004) of each best-ruleset was calculated using unweighted and weighted edges. When nodes of the same class are connected more frequently than would be expected by chance, the modularity is positive; similarly, if nodes of different classes are connected more frequently than expected by chance, the modularity is negative. Graph density (Coleman and Moré, 1983) is the proportion of edges present out of all possible edges and was calculated for each year-source graph and subgraphs of only within-class edges (e.g., aminoglycoside – aminoglycoside) and only between-class edges (e.g., aminoglycoside – macrolide). For a given node, the node degree is the number of other nodes connected to it by edges.

### False Discovery Rate

Some rules discovered with association rule mining may be false discoveries that occur by chance and do not represent true associations. Megiddo and Srikant (1998) demonstrated a resampling procedure to determine the statistical significance of association rules and minimize false discoveries (type I errors). We applied this procedure to determine the expected number of false discoveries in the pruned best-rulesets. Briefly, 100 null datasets were created for each year-source dataset by treating each antimicrobial resistance as an independent binomial random variable with parameters *n* (number of transactions in the year-source dataset) and *p* (prevalence of resistance in the year-source dataset). Association rules were mined, the rules were ranked in each null dataset by a given quality measure (confidence, lift, and the absolute value of phi), and the quality measure values at each rank were averaged across the 100 null datasets. The expected number of false discoveries for a given quality measure cut-off is the rank of the quality measure cut-off in the ranked averages. This can be expressed as a false discovery rate or percentage by dividing by the number of rules in the NARMS datasets that meet the quality measure cut-off (and multiplied by 100 if expressed as a percent). We also calculated the rules' *P*-values associated with each quality measure in a similar manner. Association rules were mined from the null datasets, the percentiles of confidence, lift and the absolute value of phi were calculated for each null dataset and averaged across the 100 null datasets. Association rules discovered in the NARMS datasets that meet a given quality measure cut-off have a *P*-value equal to or less than the percent of rules from the random null datasets that meet the same quality measure cut-off.

## Results

Each year-source dataset generated between 7331 and 43070 rules (Figure 2F), with larger datasets resulting in more rules. The distributions of rule quality measures (confidence, lift, phi, support) were similar across the datasets (Figure 2). Most rules had high confidence (Figure 2A), indicating that the antecedent nearly perfectly predicted the consequent. The confidence cut-off for pruning the rules was set at >0.75 to include rules with a reliability of at least 75%. The support of most rules was small (Figure 2E), which is consistent with the low frequency of resistance to most antimicrobials (Table 2). To avoid pruning interesting rules that involve rare resistances, the support cut-off was >0.01. Lift compares the support of a rule in a dataset to the support expected if the antecedent and consequent were independent. A lift of 1 indicates that the antecedent and consequent are independent; a lift <1 indicates a negative association and a lift > 1 is a positive association. Only 3.5% of rules across all datasets had a negative association between the antecedent and consequent (lift <1). A cut-off of lift >2 was chosen because it selects MDR patterns that occur at least twice as often as expected under independence. The phi correlation coefficient measures the strength of the association between the antecedent and consequent of the rule. The cut-off of >0.5 selects rules with a moderate to strong positive association between antimicrobial resistances. Pruning with confidence >0.75, support >0.01, lift > 2, and phi >0.5 results in 179 to 849 best-rules in each dataset (Figure 2F).

**Figure 3:**
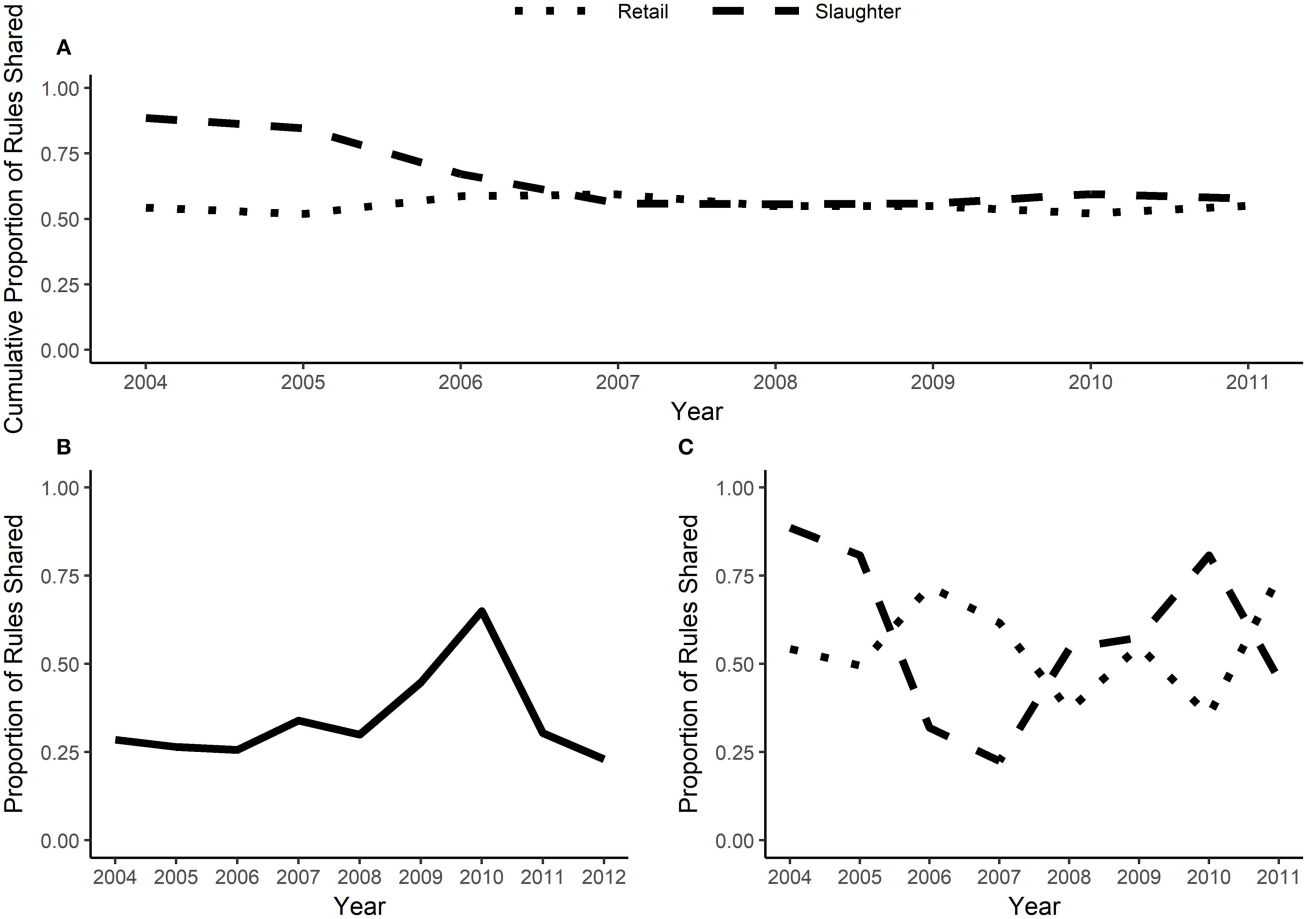
Rule overlap and cumulative rule stability. Rule overlap is the proportion of rules shared between two datasets (the number of rules that are in both datasets divided by the total number of rules within the datasets). When calculated for consecutive years, rule overlap is plotted on the earlier year. Cumulative rule stability **(A)** averages the proportion of rules shared across all previous years. **(B)** is rule overlap between slaughter and retail isolates for a given year and **(C)** is rule overlap between consecutive years, calculated separately for slaughter and retail isolates.

The best-rule sets can be compared across years and sources (retail vs. slaughter). A high rule overlap ratio indicates a high degree of similarity between two datasets; in the context of AMR it indicates that the same MDR patterns are found in two year-source datasets. In general, approximately one quarter of the best-rules in a given year were found in both the retail and slaughter isolates (Figure 3B). There was significantly more variability in rule overlap between consecutive years (Figure 3C). Between 36 and 75% of the best-rules from retail isolates overlapped with the next year; the proportion of rules shared between consecutive years in slaughter data varied from 90 to 25%. When the proportion of rules shared is averaged across all previous years, both the retail and slaughter datasets had ~50% rule overlap after four years (Figure 3A).

The average difference in support of the best-rules in retail and slaughter datasets ranged from 0.007 to 0.03 (Figure S1A), similar to the average support difference between consecutive years for both retail and slaughter data (Figure S1B). This difference is 14 to 60% of the average best-rule support (0.05). The average confidence difference for best-rules between sources (Figure S1A) and consecutive years (Figure S1B) ranges from ~0.01 to 0.04, which is only 1 to 4% of the average confidence (0.97).

**Figure 4:**
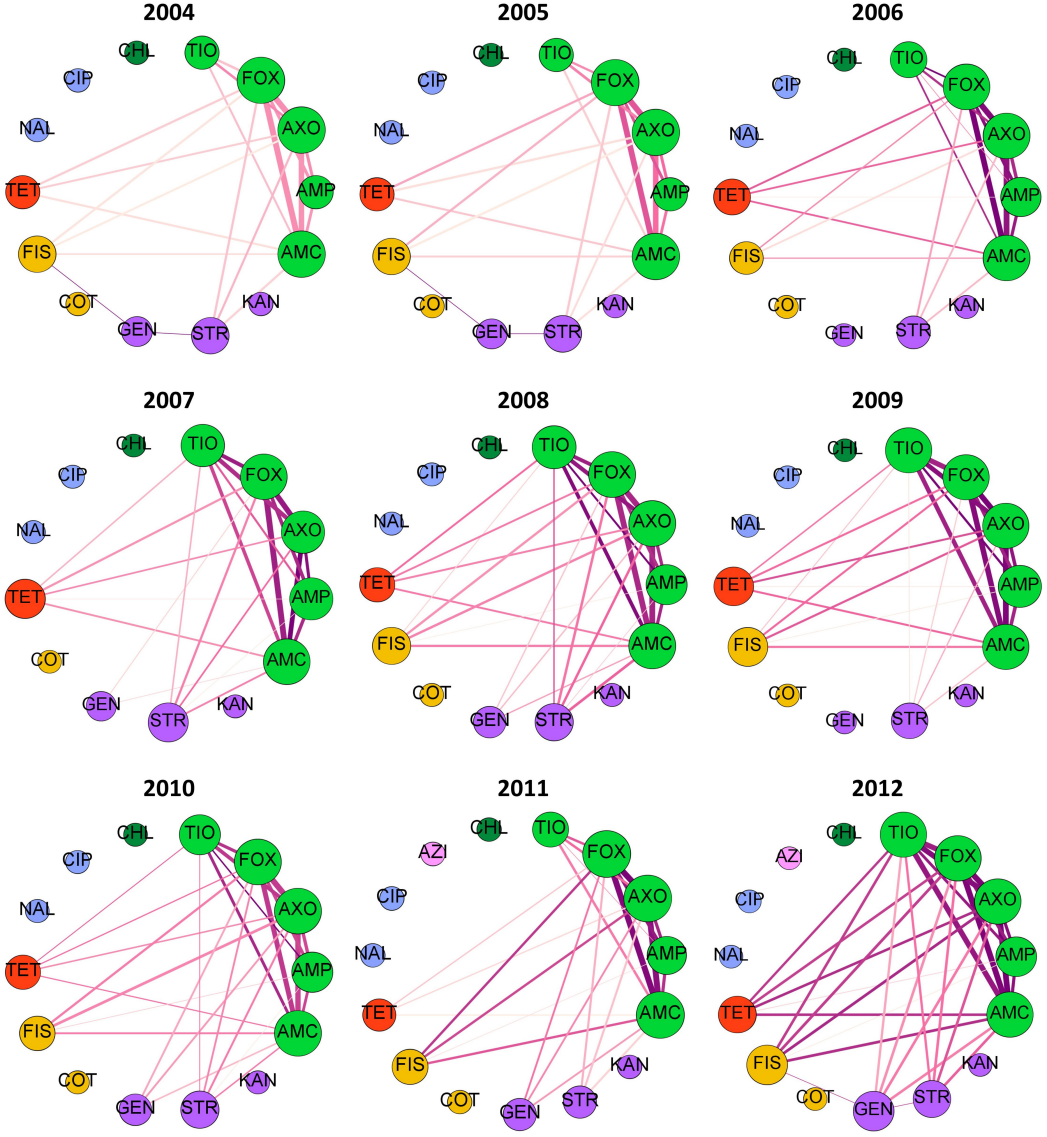
Decomposed rule graphs for *Escherichia coli* isolated from chicken carcasses at slaughter. The best-rules identified in each year (confidence >0.75, support >0.01, lift >2, phi >0.5) were decomposed into nodes (antimicrobials) and edges connecting the antecedents to the consequent. Nodes are colored based on antimicrobial class (bright green = beta-lactams; purple = aminoglycosides; yellow = sulfonamides; red = tetracycline; blue = fluoroquinolones; dark green = phenicols; pink = macrolides). Node size is proportional to node degree (number of other connected nodes). Edge thickness is proportional to the number of rules involving each pair of antimicrobials and edge darkness is proportional to the average phi for those rules.

**Figure 5:**
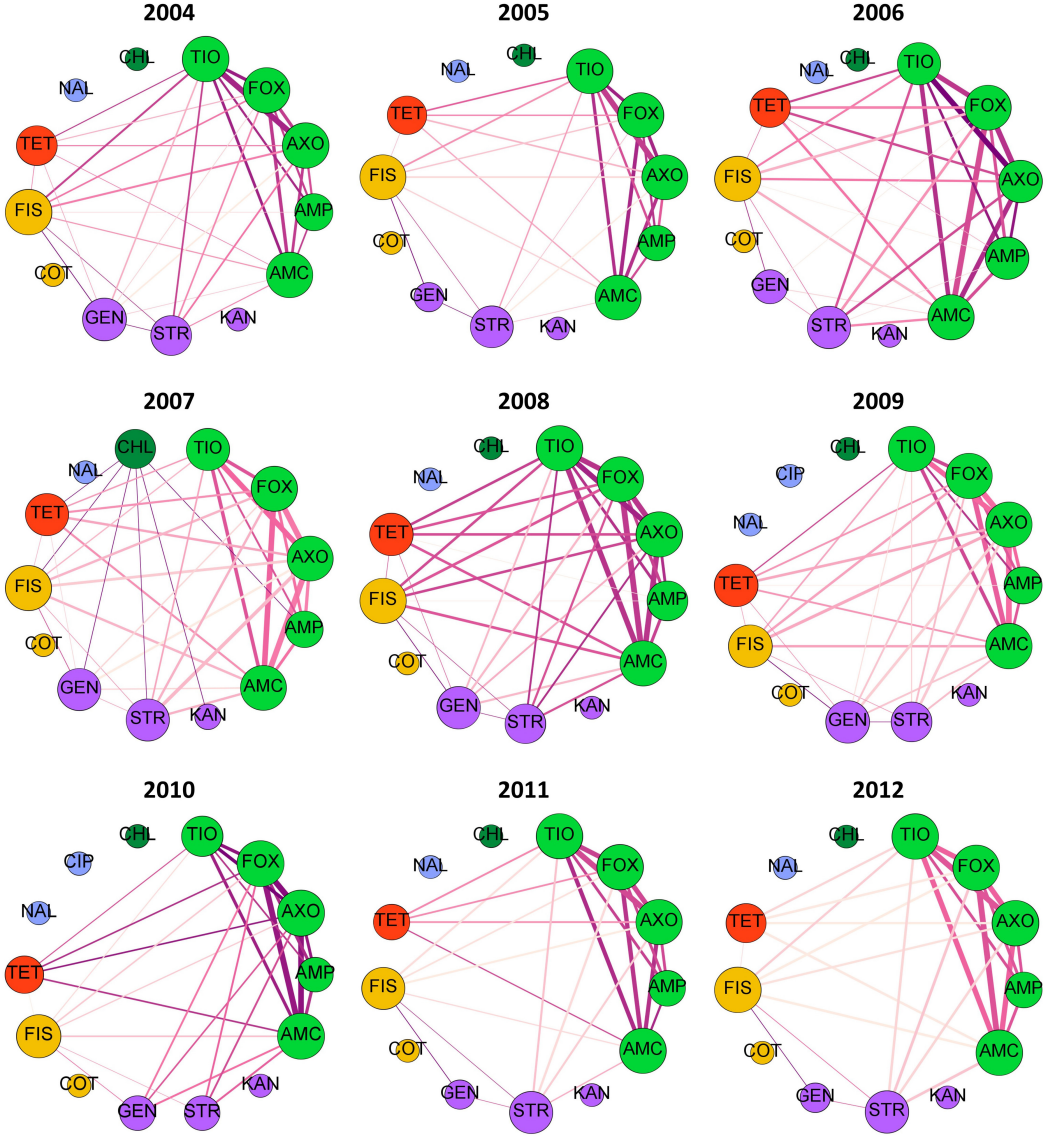
Decomposed rule graphs for *Escherichia coli* isolated from chicken retail meat. The best-rules identified in each year (confidence >0.75, support >0.01, lift >2, phi >0.5) were decomposed into nodes (antimicrobials) and edges connecting the antecedents to the consequent. Nodes are colored based on antimicrobial class (bright green = beta-lactams; purple = aminoglycosides; yellow = sulfonamides; red = tetracycline; blue = fluoroquinolones; dark green = phenicols). Node size is proportional to node degree (number of other connected nodes). Edge thickness is proportional to the number of rules involving each pair of antimicrobials and edge darkness is proportional to the average phi for those rules.

If rules are decomposed into edges and nodes, with an edge connecting each antimicrobial in the antecedent to the consequent antimicrobial, then network diagnostics can be used to evaluate the MDR patterns. Fifteen antimicrobial resistances were evaluated in each year, with the exception of 2007 slaughter isolates which were not tested against FIS. Graph density, the proportion of the 105 possible edges (pairs of antimicrobial resistances) that are found in each best-ruleset, ranged from 25 to 50% (Figure S2A). Out of the 15 possible within-class resistance edges (connecting beta-lactams, aminoglycosides, sulfonamides, or fluoroquinolones), nearly 75% were consistently found in the best rules (Figure S2A). All 10 within-beta-lactam resistance edges are typically identified with the best-rules with an occasional edge between GEN and STR (Figures 4, 5). The within-beta-lactam resistance edges occur repeatedly in the best-rulesets (high edge weight in Figures 4, 5) and also usually have the largest correlation coefficients (edge darkness in Figures 4, 5) in each best-ruleset. Only ~20 to 40% of the 90 possible between-class edges are found in a given year and source (Figure S2A). Almost all of the between-class rules connect a beta-lactam to TET, FIS, STR or GEN, with rare connections between FIS, GEN, STR, and TET (Figures 4, 5). The between-class resistance edges appear with varied frequency (edge weight) and correlation coefficients (edge darkness) in each best-ruleset (Figures 4, 5) but are always less frequent than the within-beta-lactam resistance edges. Both the weighted and unweighted modularity of each best-rule network are close to 0 (Figures S2B,C), indicating that the edges are approximately randomly distributed among the nodes (if node degree is kept constant).

**Figure 6:**
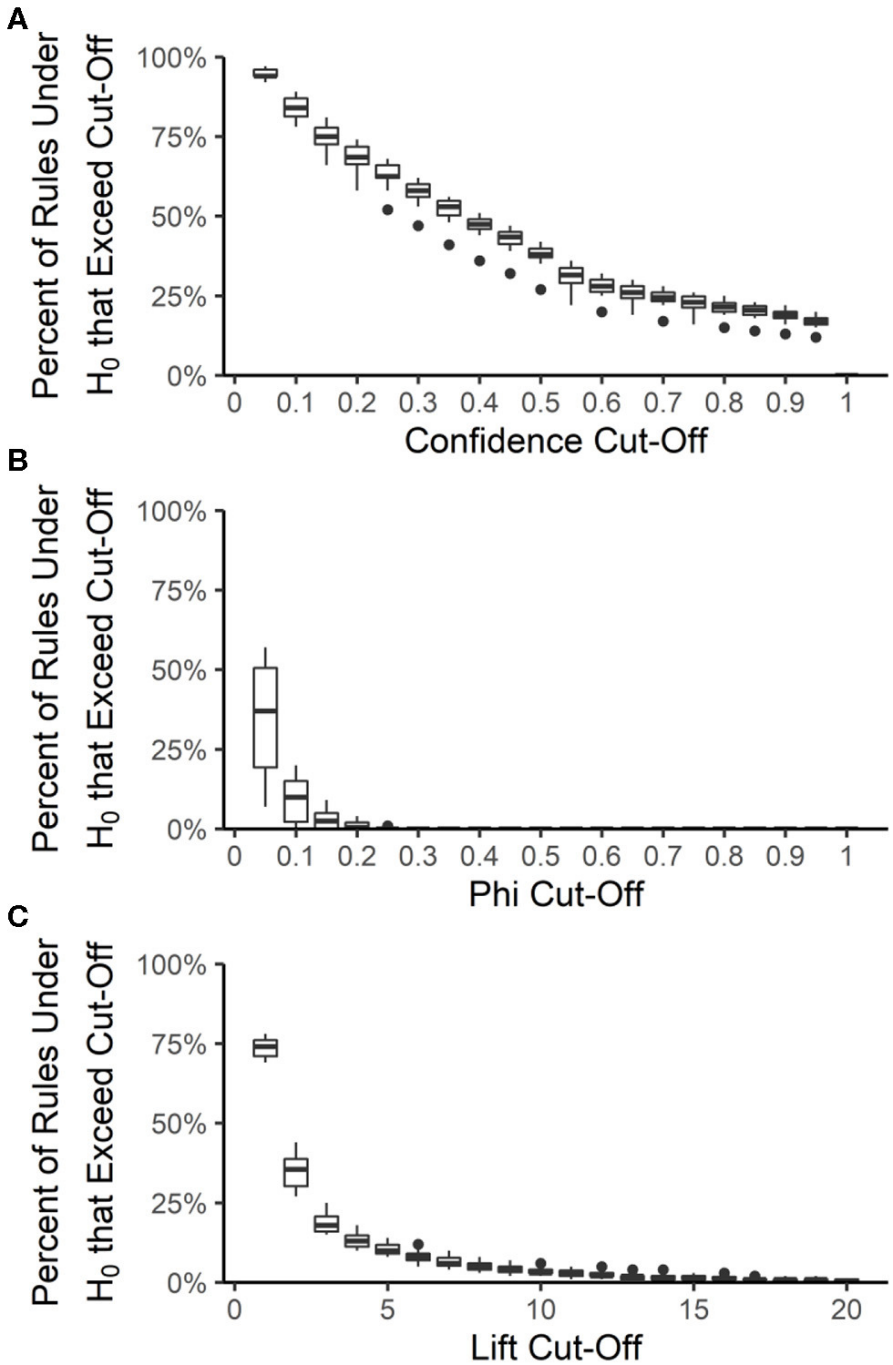
Distribution of association rule quality measures under the null hypothesis (H_0_) of no associations. The percent of rules under the null hypothesis (H_0_) that exceed a given quality measure cut-off was calculated for each year-source dataset and at 20 different cut-off values for confidence **(A)**, phi **(B)**, and lift **(C)**. Boxes are the interquartile ranges among the year-source datasets; solid line is the median, whiskers extend up to 1.5 times the interquartile range and any outliers are marked with points.

The false discovery rate among the best-rulesets and the expected rule *P*-values were calculated by creating 100 datasets from each year and source, maintaining the prevalence of each resistance but allowing each resistance to be an independent random variable. The rank and distribution of rule quality measures in the null datasets were used to determine the expected false discovery rate and expected rule *P*-values, respectively, at each quality measure value that could be used for pruning rulesets. Rule confidence (i.e., conditional probability) is not a useful quality measure for determining whether rules are true associations or false discoveries because 12 to 20% of rules under the null hypothesis of no association have a confidence >0.95 (Figure 6A). We used confidence >0.75 to prune each ruleset to the best-ruleset; 16 to 26% of rules under the null hypothesis meet this cut-off. We also removed rules with lift ≤2; 27 to 44% of rules under the null hypothesis meet this cut-off. Lift >10 is required to achieve a *P*-value of ≤0.05 (Figure 6C). The absolute value of phi can be as small as 0.2 and still result in a *P*-value ≤ 0.05 (Figure 6B). Our best-rules had phi >0.5, which was associated with an expected *P*-value of <0.01. Accounting for the number of rules in the NARMS datasets that meet each of these quality-measure cut-offs, the maximum expected false discovery rate associated with confidence >0.75 is 11%, with lift >2 is 13%, and with phi >0.5 is 0.4%. Therefore, the combination of confidence >0.75, lift >2, and phi >0.5 that was used to create the best rule-sets is expected to result in <1% false discoveries, under the assumption of independent drug resistances.

## Discussion

The patterns of multidrug resistance in NARMS isolates have been explored with several statistical techniques, including log-linear models (Zawack et al., 2018), Bayesian networks (Zawack et al., 2018), and Markov networks (Love et al., 2016, 2018). Although other machine learning techniques (e.g., decision trees, hierarchical clustering) have been applied to resistance datasets (Coelho et al., 2013), association rule mining has not been investigated as a method for identifying patterns and trends of multiple antimicrobial resistances. Traditional statistical techniques may be limited by the non-Gaussian and sparse nature of AMR surveillance data; rule mining overcomes these limitations because it does not make assumptions about the data distribution. In addition, rule mining identifies complex high-order associations (up to eight-way interactions in this data, Table S3) because it searches the entire MDR pattern space. In contrast, Markov networks examine pairwise interactions (Love et al., 2016) and Bayesian networks identified up to four-way interactions in the NARMS chicken *E. coli* data (Zawack et al., 2018).

The Apriori algorithm can efficiently process datasets with many isolates and tested antimicrobials. However, thousands of associations can be identified from relatively small datasets and must be appropriately pruned in order to find interesting patterns that represent true associations in the data (Tan et al., 2018). By implementing a quality measure selection pipeline (Martínez-Ballesteros et al., 2014) using PCA, we identified the rule quality measures that had the greatest orthogonal variability in the NARMS datasets. Confidence, lift, phi and support measure distinct aspects of associations: conditional probability, independence, correlation, and frequency, respectively. However, they should be interpreted in tandem because individual quality measures can be adversely affected by skewed resistance distributions (Hahsler, 2015; Tan et al., 2018). The *E. coli* isolated from chicken at slaughter and retail meats between 2004 and 2012 are infrequently resistant to many of the tested antimicrobials (Table 2). This limits the utility of support as a pruning quality measure and reduces the efficiency of the Apriori algorithm for finding association rules (Agrawal et al., 1993; Tan et al., 2018). When some resistances are rare and others are frequent, rule confidence can be high even when the resistances are independent (Tan et al., 2018). Hence a skewed resistance distribution also impacts the ability to evaluate rules using conditional probability. This is reflected in the poor performance of confidence in the false discovery rate resampling procedure. By pairing confidence and support with lift and phi, we were able to identify patterns of antimicrobial resistance that were dependent (lift and phi), common (support), and reliable (confidence).

The resampling procedure demonstrated that the expected false discovery rate was <1% in the NARMS datasets when examining rules with confidence >0.75, lift >2, and phi >0.5; this is specific to the tested datasets because rule quality measures can be affected by changes in resistance prevalence and sample size (e.g., phi is sensitive to scaling within the antecedent-consequent contingency table) (Tan et al., 2004). Therefore, we are confident that the patterns of antimicrobial resistance uncovered after rule pruning likely represent true associations present in these datasets. However, these associations may not be present in other datasets and may not reflect the patterns of antimicrobial resistance in the broader population of *E. coli* associated with chicken. NARMS sampling strategies for slaughter and processing plants changed in 2006 from combined random (non-targeted) and targeted sampling to risk-based sampling and then changed again in 2013 to random cecal samples (National Antimicrobial Resistance Monitoring System, 2016b). Retail samples have been randomly selected since 2002 although not all states are represented in the retail sampling network (National Antimicrobial Resistance Monitoring System, 2016b). Hence the NARMS datasets used in this study are likely a biased representation of all *E. coli*-contaminated chicken and latent variables may mask or confound patterns of multidrug resistance. This is supported by the significant over dispersion found in antimicrobial resistance contingency tables from the same *E. coli* isolates (Zawack et al., 2019).

The over dispersion and latent variables likely contribute to the variable rule overlap and moderate cumulative rule stability (Figure 3) in the NARMS datasets. If sufficient metadata is published with isolate susceptibility results, then potential confounding variables can be included in the association rules and rule overlap may have greater potential for detecting true temporal changes in MDR patterns. Since we used the best-rulesets with a <1% false discovery rate to calculate rule overlap, type II errors (false negatives) may also contribute to the variability in overlap between consecutive years and the low overlap between slaughter and retail. Relaxing the false discovery rate will increase the power to find all significant associations at the expense of having some false discoveries. The low rule overlap between slaughter and retail isolates is not due to differences in dataset size (Figure S3). The low rule overlap may be driven by the associations between GEN—STR, TET—FIS, and FIS—GEN, which are frequently present in retail isolate best-rules and mostly absent in slaughter isolate best-rules (Figures 4, 5). These edges are present in the slaughter rules before pruning and often have a moderate phi (>0.2) but are lower than the lift and confidence pruning cut-offs. Therefore, these rules may be false negatives in the pruning procedure and adjusted cut-offs may increase the rule overlap between slaughter and retail. The cause of increased rule overlap between slaughter and retail in 2010 (Figure 3B; Figure S3) is uncertain. We are not aware of a change in NARMS sampling strategy in 2010 that may have resulted in greater similarity between the slaughter and retail sampled populations (Karp et al., 2017). One possibility is an outbreak of *E. coli* that originated in chicken carcasses, resulting in a high prevalence of clonally-related isolates on retail chicken. However, there was not a reported chicken-associated *E. coli* outbreak in 2010 (Centers for Disease Control and Prevention, 2015).

If the strength of a MDR pattern (caused by genetic linkage or a common mechanism) does not change significantly over-time, then the confidence of a MDR association rule should remain relatively stable and the support of the rule may change over time as the MDR pattern becomes more prevalent. Examining differences in rule quality between the datasets demonstrates that the best-rules have consistent reliability (Figure S1), with very small changes in confidence year to year and between slaughter and retail. However, the support of the best-rules changes significantly (Figure S1) as a percentage of average support. The large changes in rule support could reflect true differences in the prevalence of MDR patterns year to year and between slaughter and retail sources. More likely, high variance of multidrug resistance patterns in the target population of *E. coli* and relatively small sample sizes results in large variations in MDR pattern prevalence in the datasets. The relatively small changes in confidence suggest that the shared MDR patterns are not changing substantially in the target population. The overall prevalence of MDR in *E. coli* has increased significantly over the last 70 years (Tadesse et al., 2012); applying association rule mining to surveillance data over a long time-period may elucidate the emergence and spread of specific MDR patterns.

Multidrug resistance pattern stability is also supported by the consistent resistance subnetworks of beta-lactams, tetracycline, sulfisoxazole, and aminoglycosides (Figures 4, 5). Associations between beta-lactam resistances have been identified from NARMS chicken *E. coli* MIC data using Markov networks (Love et al., 2016) and from binary resistance data using Bayesian networks (Zawack et al., 2018). Resistance to AMP, AMC, FOX, TIO, and AXO was one of the most common MDR patterns found in chicken-associated *E. coli* in Canada (MacKinnon et al., 2018). Resistance to multiple beta-lactams is frequently mediated through cross-resistance (e.g., efflux pumps or beta-lactamase production) but isolates may also have multiple beta-lactam resistance genes (co-resistance) (King et al., 2017). The relatively low number of edges and low-weight of edges connected to AMP compared to the other beta-lactams (Figures 4, 5) suggests that at least two different mechanisms or beta-lactam resistance genes are present in this population of *E. coli* because genes that confer resistance against newer beta-lactams should also confer resistance to AMP. For example, many *E. coli* may carry a broad-spectrum beta-lactamase (e.g., TEM-1, SHV-1) that hydrolyzes penicillin and ampicillin but not cephalosporins (FOX, TIO, AXO) and is inhibited by clavulanic acid (AMC) (Paterson and Bonomo, 2005; Paterson and Doi, 2017). Other isolates may have AmpC beta-lactamases, which confer resistance to almost all beta-lactams and beta-lactamase inhibitors (Paterson and Bonomo, 2005; Paterson and Doi, 2017). The circulation of different beta-lactam resistance genes is also supported by the higher prevalence of resistance to AMP than resistance to other beta-lactams (Table 2). The consistent beta-lactam edges in the slaughter and retail rule networks suggest the continuous presence of beta-lactam resistance genes in *E. coli* on chicken carcasses and retail meats.

The relationships observed between beta-lactams, aminoglycosides, sulfisoxazole and tetracycline resistances could occur with multi-drug resistance efflux pumps (cross-resistance), although few resistance pumps confer resistance to all of those antimicrobial classes (Li, 2017). Resistance genes to these drugs may be genetically linked if they are incorporated into the same mobile genetic element or an association may develop through the sequential or simultaneous use of different antimicrobials (Chang et al., 2015). In Canada, distinct MDR patterns involving one or more of TET, STR, and FIS, plus either just AMP or all five beta-lactams, have been recorded in chicken-associated *E. coli* (MacKinnon et al., 2018). Both human- and animal-associated *E. coli* isolated in the U.S. between 1950 and 2002 also have MDR patterns containing TET, STR, and FIS plus just AMP or AMP, AMC, and cephalosporins (Tadesse et al., 2012). The association rules involving AMP and FIS, TET, STR or GEN are relatively weak and infrequent compared to the rules with cephalosporins and other antimicrobial classes (Figures 4, 5). This suggests that the co-resistance between AmpC or extended-spectrum beta-lactamases and other antimicrobial class resistance genes may be more substantial than co-resistance between beta-lactamase genes like TEM-1 and other resistance genes.

Markov networks identified a subnetwork involving aminoglycosides, sulfonamides, and tetracycline resistances and pairwise associations between beta-lactam resistances and these drug classes were revealed with small LASSO penalties (Love et al., 2016). Bayesian networks revealed a similar pattern, with ampicillin connecting a beta-lactam resistance network to an aminoglycoside-sulfonamide-tetracycline resistance network (Zawack et al., 2018). MDR patterns involving GEN, FIS, STR, and/or TET (without beta-lactams) were identified in Canadian chicken-associated *Escherichia coli* (MacKinnon et al., 2018) and in older U.S. *E. coli* isolated from animals and humans (Tadesse et al., 2012). The association rule decomposed graphs do not contain a separate aminoglycoside-sulfonamide-tetracycline resistance subnetwork but rather connect FIS, GEN, STR and TET resistances individually to the beta-lactam resistance subnetwork. Many individual best-rules do contain those antimicrobials within the same antecedent (Table S3), however the decomposed graphs only connect each individual antecedent to the consequent in order to reduce graph density. Since these drugs only appear with beta-lactams in the best-rules, the prevalence of GEN-STR-TET-FIS MDR patterns in other datasets (Tadesse et al., 2012; MacKinnon et al., 2018) may reflect random chance with high individual resistance prevalences rather than a strong co-resistance.

In the United States, several antimicrobial classes are approved for use in broiler chickens (Food Animal Residual Avoidance and Depletion Program, 2018): macrolides, aminoglycosides, aminocoumarins, orthosomycins, polypeptides, bambermycins, tetracyclines, lincosamides, sulfonamides, and streptogramins. Aminoglycosides and macrolides are considered critically important for human health, and lincosamides, sulfonamides, streptogramins, and tetracyclines are highly important (World Health Organization, 2016). Orthosomycins, aminocoumarins, and bambermycins are not currently used in human health (World Health Organization, 2016). Even though beta-lactams are not approved for use in broilers, the association rules involving beta-lactam resistances and sulfonamide, tetracycline and aminoglycoside resistances demonstrate that the prevalence of beta-lactam resistance could increase in chicken-associated *E. coli* through the use of other approved drugs.

Association rule mining can be applied to any antimicrobial susceptibility dataset with more than a few hundred isolates; the smallest dataset in this analysis had 299 isolates (Table 2). This technique is particularly useful when a large number of antimicrobials are tested because the number of possible MDR patterns increases exponentially with the number of tested drugs. Association rule mining has been previously applied to antimicrobial resistance as a means for detecting infection outbreaks in hospitals (Ma et al., 2003; Tsymbal, 2005; Giannopoulou et al., 2007; Gerontini et al., 2011). Rules for this task contain significant amounts of metadata on the hospital, clinical department, and patient with a drug resistance or bacterial organism in the consequent. These studies examined leverage (Giannopoulou et al., 2007; Gerontini et al., 2011), confidence (Gerontini et al., 2011), and expert opinion (Ma et al., 2003; Tsymbal, 2005) to determine which rules were interesting and when outbreaks may have occurred. In contrast, our approach focuses on including only drug resistances in the rules in order to detect MDR patterns. We used principal component analysis to identify the rule quality measures that explain the largest amount of variability in the rules and therefore we were able to select the strongest, most interesting rules. The same approach can be applied to clinical data to detect MDR patterns in nosocomial or community infections. Metadata associated with clinical isolates can be incorporated into the MDR rules as either an antecedent or consequent to identify local resistance trends or outbreaks within hospitals. Recommendations for antimicrobial stewardship or infection control could be developed from such association rules (Ma et al., 2003; Giannopoulou et al., 2007).

In conclusion, association rule mining is an effective tool for identifying patterns of multidrug resistance within antimicrobial susceptibility testing data and evaluating the statistical and biological significance of the patterns. Rule quality measures used to sort and differentiate rules should be tested using resampling procedures to minimize the false discovery rate. Rule mining identified consistent multidrug resistance patterns involving beta-lactams, sulfisoxazole, tetracycline, gentamicin, and streptomycin in *E. coli* isolated from chicken carcasses and meat between 2004 and 2012. The generally low rule overlap suggests that over dispersion or latent variables result in considerable variability in rule composition. In contrast, the stable density and common edges in the decomposed graphs imply that the underlying associations between drug resistances in the chicken-associated *E. coli* population did not change significantly over time.

## Author Contributions

CC developed the analytic pipeline, implemented the rule mining analysis, contributed to the interpretation of results, and prepared the manuscript. MA-M conceived of and designed the project, contributed to the analytic pipeline development and interpretation of results, and revised the manuscript. KK contributed to the analytic pipeline development, implementation of rule mining, and interpretation of results, and revised the manuscript. WL contributed to data preparation, analytic pipeline development, interpretation of results, and revised the manuscript. JB contributed to the false discovery rate experiment design, analytic pipeline development, interpretation of results, and revised the manuscript. CL contributed to analytic pipeline development, interpretation of results, and revised the manuscript. YG contributed to the design of the project, analytic pipeline development, interpretation of results, and revised the manuscript.

### Conflict of Interest Statement

The authors declare that the research was conducted in the absence of any commercial or financial relationships that could be construed as a potential conflict of interest.

## Abbreviations

AMR: antimicrobial resistance
MDR: multidrug resistance
AMP: ampicillin
AMC: amoxicillin-clavulanic acid
AXO: ceftriaxone
FOX: cefoxitin
TIO: ceftiofur
AMI: amikacin
GEN: gentamicin
KAN: kanamycin
STR: streptomycin
COT: trimethoprim-sulfamethoxazole
FIS: sulfisoxazole
CIP: ciprofloxacin
NAL: nalidixic acid
TET: tetracycline
CHL: chloramphenicol
AZI: azithromycin.
