# Corrigendum: Shared Multidrug Resistance Patterns in Chicken-Associated *Escherichia coli* Identified by Association Rule Mining

**DOI:** 10.3389/fmicb.2020.01359

**Published:** 2020-06-30

**Authors:** Casey L. Cazer, Mohammad A. Al-Mamun, Karun Kaniyamattam, William J. Love, James G. Booth, Cristina Lanzas, Yrjö T. Gröhn

**Affiliations:** ^1^Department of Population Medicine and Diagnostic Sciences, Cornell University College of Veterinary Medicine, Ithaca, NY, United States; ^2^Department of Epidemiology of Microbial Diseases, Yale University School of Public Health, New Haven, CT, United States; ^3^Department of Population Health and Pathobiology, North Carolina State University College of Veterinary Medicine, Raleigh, NC, United States; ^4^Department of Biological Statistics and Computational Biology, Cornell University College of Agriculture and Life Sciences, Ithaca, NY, United States

**Keywords:** association rule mining, antimicrobial resistance, *Escherichia coli*, machine learning, multidrug resistance, foodborne bacteria

In the original article, there was an error in the description and presentation of the false discovery rate. As written, the false discovery rate methodology describes a *P*-value calculation under a null hypothesis (H0) of no association between the individual antimicrobial resistances. However, we did assess the false discovery rate using the procedure described by Megiddo and Srikant (1). This procedure ranks the rules in each null dataset by a given quality measure and averages the quality measure value across the 100 null datasets at each rank. The two methodologies were conflated in the manuscript. It was correctly stated in the Results and Discussion section that the combination of confidence > 0.75, lift > 2, and phi > 0.5 will result in < 1% false discovery rate, as measured by the Megiddo and Srikant method (1998). We have since confirmed this false discovery rate of < 1% by counting the number of rules from each of the 100 random null datasets that meet these three quality measure cut-offs. We estimated the expected number of false discoveries as the average of these counts across the 100 random null datasets and expressed this as a percent of the NARMS dataset rules that meet the three quality measure cut-offs. We encourage authors seeking to use a similar false discovery rate procedure to reference Megiddo and Srikant (1). Code used to create the analysis is available from the authors upon request. The correction has been made to the **Methods** section, False Discovery Rate sub-section and the **Results** section, Paragraph 5, respectively:

Some rules discovered with association rule mining may be false discoveries that occur by chance and do not represent true associations. Megiddo and Srikant (1998) demonstrated a resampling procedure to determine the statistical significance of association rules and minimize false discoveries (type I errors). We applied this procedure to determine the expected number of false discoveries in the pruned best-rulesets. Briefly, 100 null datasets were created for each year-source dataset by treating each antimicrobial resistance as an independent binomial random variable with parameters *n* (number of transactions in the year-source dataset) and *p* (prevalence of resistance in the year-source dataset). Association rules were mined, the rules were ranked in each null dataset by a given quality measure (confidence, lift, and the absolute value of phi), and the quality measure values at each rank were averaged across the 100 null datasets. The expected number of false discoveries for a given quality measure cut-off is the rank of the quality measure cut-off in the ranked averages. This can be expressed as a false discovery rate or percentage by dividing by the number of rules in the NARMS datasets that meet the quality measure cut-off (and multiplied by 100 if expressed as a percent). We also calculated the rules' *P*-values associated with each quality measure in a similar manner. Association rules were mined from the null datasets, the percentiles of confidence, lift and the absolute value of phi were calculated for each null dataset and averaged across the 100 null datasets. Association rules discovered in the NARMS datasets that meet a given quality measure cut-off have a *P*-value equal to or less than the percent of rules from the random null datasets that meet the same quality measure cut-off.

The false discovery rate among the best-rulesets and the expected rule *P*-values were calculated by creating 100 datasets from each year and source, maintaining the prevalence of each resistance but allowing each resistance to be an independent random variable. The rank and distribution of rule quality measures in the null datasets were used to determine the expected false discovery rate and expected rule *P*-values, respectively, at each quality measure value that could be used for pruning rulesets. Rule confidence (i.e., conditional probability) is not a useful quality measure for determining whether rules are true associations or false discoveries because 12 to 20% of rules under the null hypothesis of no association have a confidence >0.95 (Figure 6A). We used confidence >0.75 to prune each ruleset to the best-ruleset; 16 to 26% of rules under the null hypothesis meet this cut-off. We also removed rules with lift ≤2; 27 to 44% of rules under the null hypothesis meet this cut-off. Lift >10 is required to achieve a *P*-value of ≤0.05 (Figure 6C). The absolute value of phi can be as small as 0.2 and still result in a *P*-value ≤ 0.05 (Figure 6B). Our best-rules had phi >0.5, which was associated with an expected *P*-value of <0.01. Accounting for the number of rules in the NARMS datasets that meet each of these quality-measure cut-offs, the maximum expected false discovery rate associated with confidence >0.75 is 11%, with lift >2 is 13%, and with phi >0.5 is 0.4%. Therefore, the combination of confidence >0.75, lift >2, and phi >0.5 that was used to create the best rule-sets is expected to result in <1% false discoveries, under the assumption of independent drug resistances.

**Figure 6:**
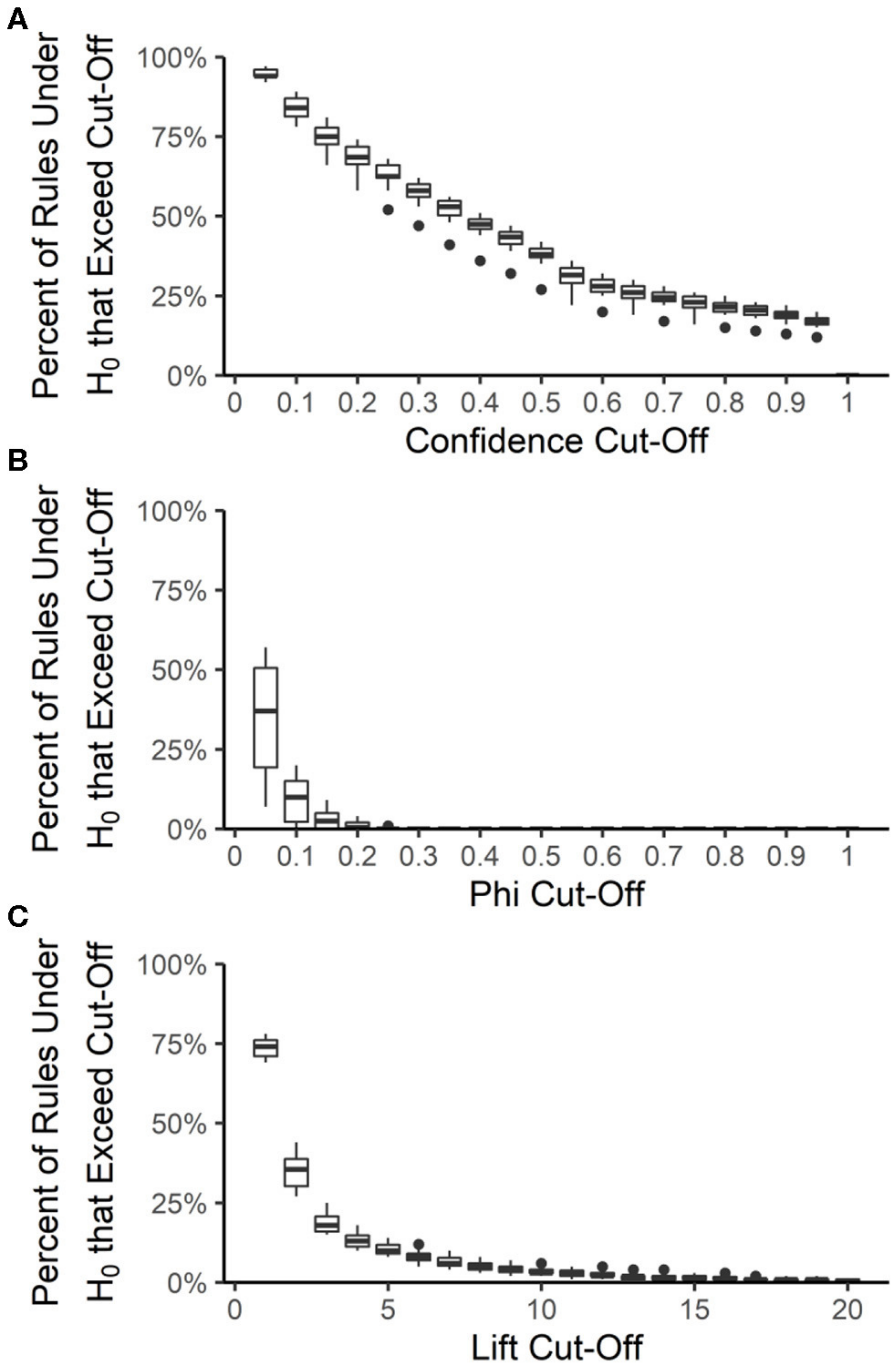
Distribution of association rule quality measures under the null hypothesis (H_0_) of no associations. The percent of rules under the null hypothesis (H_0_) that exceed a given quality measure cut-off was calculated for each year-source dataset and at 20 different cut-off values for confidence **(A)**, phi **(B)**, and lift **(C)**. Boxes are the interquartile ranges among the year-source datasets; solid line is the median, whiskers extend up to 1.5 times the interquartile range and any outliers are marked with points.

Similarly, in the original article, the y-axis label and legend of Figure 6 are incorrect. Figure 6 presents the results from the procedure describing *P*-value calculations and not the Megiddo and Srikant (1998) false discovery rate procedure. The corrected Figure 6 and legend appears below.

We also noticed that the stated number of isolates tested in the Abstract and Methods (*n* = 21,243) incorrectly included isolates tested between 2000 and 2003 plus 2013. The total isolates tested between 2004 and 2012 is 14,418 as correctly reported in Table 2. The correction has been made to the Abstract and the Methods section, Data Sources sub-section, respectively:

Using multiple antimicrobials in food animals may incubate genetically-linked multidrug-resistance (MDR) in enteric bacteria, which can contaminate meat at slaughter. The U.S. National Antimicrobial Resistance Monitoring System tested 14,418 chicken-associated *Escherichia coli* between 2004 and 2012 for resistance to 15 antimicrobials, resulting in >32,000 possible MDR patterns. We analyzed MDR patterns in this dataset with association rule mining, also called market-basket analysis. The association rules were pruned with four quality measures resulting in a <1% false-discovery rate. MDR rules were more stable across consecutive years than between slaughter and retail. Rules were decomposed into networks with antimicrobials as nodes and rules as edges. A strong subnetwork of beta-lactam resistance existed in each year and the beta-lactam resistances also had strong associations with sulfisoxazole, gentamicin, streptomycin and tetracycline resistances. The association rules concur with previously identified *E. coli* resistance patterns but provide significant flexibility for studying MDR in large datasets.

Antimicrobial susceptibility testing data from *Escherichia coli* isolated from chicken carcasses since 2000 and from retail chicken meat since 2002 as part of NARMS surveillance is publicly available (Food and Drug Administration, 2016). Data from 2004 to 2012 (14,418 isolates) were used for this study because of changes in NARMS sampling strategies (Karp et al., 2017) and for consistency with previous studies of AMR associations in NARMS isolates (Love et al., 2016). Each isolate was tested against 12 to 25 antimicrobial drugs using the Sensititre system (National Antimicrobial Resistance Monitoring System, 2016a). The MIC results of the 15 most commonly tested antimicrobials plus azithromycin were used for this study (Table 1). Each isolate was classified as resistant or susceptible based on published MIC breakpoints (Love et al., 2016; National Antimicrobial Resistance Monitoring System, 2017). Resistance data were separated by year and source (slaughter and retail) into 18 datasets for association rule mining. The prevalence of resistance against the 16 included antimicrobials in each year-source dataset is given in Table 2.

The authors apologize for these errors and state that this does not change the scientific conclusions of the article in any way. The original article has been updated.
