# Supplementary material for: Shared Multidrug Resistance Patterns in Chicken-Associated Escherichia coli Identified by Association Rule Mining
Source: Front Microbiol. 2019 Apr 12;10:687. doi: 10.3389/fmicb.2019.00687 (PMC6473086; doi:10.3389/fmicb.2019.00687)

Table S1: Rule quality measures available in the R package ‘arules’ with equations and interpretations. Abbreviations: rule consequent (Y), rule antecedent (X), confidence (conf), support (sup). Equation References: (Tan et al., 2004; Hahsler, 2015)

| Quality Measure | Equation | Interpretation |
| --- | --- | --- |
| Added Value | $conf(X\to Y)-sup(Y)=\frac{P\left(X\cap Y\right)-P\left(X\right)P\left(Y\right)}{P\left(X\right)}$ | The difference in the support for the rule X → Y and the expected support if X and Y are independent, compared to the support of X. |
| Chi-Squared | $\sum\frac{\left(O-E\right)^{2}}{E}$ | Chi-squared statistic for testing the independence of the consequent and antecedent of a rule, calculated from the observed (O) and expected (E) values of a consequent/antecedent contingency table. |
| Certainty Factor | $\frac{conf(X\to Y)-sup(Y)}{1-sup(Y)}=\frac{P\left(Y\vert X\right)-P\left(Y\right)}{1-P\left(Y\right)}$ | The variation in the probability that Y is in transactions that contain X. |
| Collective Strength | $\frac{P\left(X\cap Y\right)+P\left(\bar{Y}\vert\bar{X}\right)}{P\left(X\right)P\left(Y\right)+P\left(\bar{X}\right)P\left(\bar{Y}\right)}$ | Equals 0 when X and Y are perfectly negatively correlated, infinity when they are perfectly positively correlated, and 1 when they are independent. |
| Confidence | $conf(X\to Y)=\frac{P\left(X\cap Y\right)}{P\left(X\right)}=P\left(Y\vert X\right)$ | The conditional probability of Y given X. |
| Conviction | $\frac{1-sup(Y)}{1-conf(X\to Y)}=\frac{P\left(X\right)P\left(\bar{Y}\right)}{P\left(X\cap\bar{Y}\right)}$ | The ratio of how frequently an incorrect prediction would be made if X and Y were independent to how frequently an incorrect prediction occurs in the dataset. |
| Cosine | $\frac{P\left(X\cap Y\right)}{\sqrt{P\left(X\right)P\left(Y\right)}}=\sqrt{P\left(X\vert Y\right)P\left(Y\vert X\right)}$ | Equals 1 when X and Y always occur together and 0 when X and Y never occur together. |
| Count | $c_{XY}$ | The number of transactions that include the rule X → Y. |
| Coverage | $sup\left(X\right)=P\left(X\right)$ | The support of the antecedent. |
| Descriptive Confirmed Confidence | $conf(X\to Y)-conf(X\to\bar{Y})=P\left(Y\vert X\right)-P\left(\bar{Y}\vert X\right)$ | The difference between the conditional probability of Y given X and not Y given X. |
| Causal Confidence | $\begin{matrix} \frac{1}{2}\left[conf(X\to Y)+conf(\bar{X}\to\bar{Y})\right] \\ =\frac{1}{2}\left[P\left(Y\vert X\right)+P\left(\bar{Y}\vert\bar{X}\right)\right] \end{matrix}$ | The average confidence of the rule X → Y and the rule not X → not Y. |
| Causal Support | $\begin{matrix} sup\left(X\cup Y\right)+sup\left(\bar{X}\cup\bar{Y}\right)=P\left(X\cap Y\right)+P\left(\bar{X}\cap\bar{Y}\right) \\ =1-P\left(X\right)-P\left(Y\right)+2P\left(X\cap Y\right) \end{matrix}$ | The sum of the support for the rule X → Y and the rule not X → not Y. |
| Counter-Example Rate | $\frac{sup(X\cup Y)-sup(X\cup\bar{Y})}{sup(X\cup Y)}=\frac{P\left(X\cap Y\right)-P\left(X\cap\bar{Y}\right)}{P\left(X\cap Y\right)}$ | The difference of the support for the rule X → Y and the rule X → not Y, compared to the support for the rule X → Y. |
| Descriptive Confirm | $\begin{matrix} sup\left(X\cup Y\right)-sup\left(X\cup\bar{Y}\right)=P\left(X\cap Y\right)-P\left(X\cap\bar{Y}\right) \\ =2P\left(X\cap Y\right)-P\left(X\right) \end{matrix}$ | The difference of the support for the rule X → Y and the rule X → not Y. |
| Difference of Confidence | $conf(X\to Y)-conf(\bar{X}\to Y)=P\left(Y\vert X\right)-P\left(Y\vert\bar{X}\right)$ | The difference between the conditional probability of Y given X and Y given not X. |
| Fisher’s Exact Test | $P\left(C_{XY}\geq c_{XY}\right)$ | The p-value of a Fisher’s exact test of the independence of the consequent and antecedent of a rule, calculated from the observed$\left(c_{XY}\right)$and expected $\left(C_{XY}\right)$counts. |
| Gini Index | $\begin{matrix} P(X)\left[P(Y\vert X)^{2}+P(\bar{Y}\vert X)^{2}\right] \\ +P(\bar{X})\left[P(Y\vert\bar{X})^{2}+P(\bar{Y}\vert\bar{X})^{2}\right]-P(Y)^{2}-P(\bar{Y})^{2} \end{matrix}$ | A measure of quadratic entropy, related to the variance of a probability distribution. |
| Hyper Lift | $\frac{c_{XY}}{Q_{\delta}\left[C_{XY}\right]}$ | The ratio of the observed count to the highest count expected at least δ% of the time. ($Q_{\delta}$is the δ quantile of the hypergeometric distribution with parameters $c_{X},c_{Y}$). If the antecedent and consequent are independent, the hyper-lift will exceed 1 only (1-δ)% of the time. |
| Hyper Confidence | $1-P\left[C_{XY}\geq c_{XY}\vert c_{X},c_{Y}\right]$ | The probability of getting a count less than the observed count given the marginal counts $c_{X},c_{Y}$(based on the hypergeometric distribution). |
| Imbalance Ratio | $\begin{matrix} \frac{\left\vert conf(Y\to X)-conf(X\to Y)\right\vert}{conf(Y\to X)+conf(X\to Y)-conf(Y\to X)conf(X\to Y)} \\ =\frac{\left\vert P\left(X\vert Y\right)-P\left(Y\vert X\right)\right\vert}{P\left(X\vert Y\right)+P\left(Y\vert X\right)-P\left(X\vert Y\right)P\left(Y\vert X\right)} \\ =\frac{\left\vert P\left(X\right)-P\left(Y\right)\right\vert}{P\left(X\cup Y\right)} \end{matrix}$ | Measures the amount of imbalance between X and Y. Equals 0 when the conditional probability of Y given X and X given Y are the same and is close to 1 if the probabilities are very different. |
| Implication Index | $\sqrt{N}\frac{sup\left(X\cup\bar{Y}\right)-sup\left(X\right)sup\left(\bar{Y}\right)}{\sqrt{sup\left(X\right)sup\left(\bar{Y}\right)}}=\frac{c_{X\bar{Y}}-\frac{c_{X}c_{\bar{Y}}}{n}}{\sqrt{\frac{c_{X}c_{\bar{Y}}}{n}}}$ | A variation of Lerman similarity index. |
| Importance | $\log_{10}\left[\frac{L\left(X\to Y\right)}{L\left(X\to\bar{Y}\right)}\right]=\log_{10}\left[\frac{c_{XY}+1}{c_{X\bar{Y}}+1}\right]$ | The log likelihood of the consequent given the antecedent. L is the Laplace corrected confidence. |
| Improvement | $\min_{X'\subset X}\left[conf(X\to Y)-conf(X'\to Y)\right]$ | The smallest difference between the confidence of a rule and the confidence of any sub-rule, where X’ is a subset of the antecedent X. |
| Jaccard Coefficient | $\frac{sup(X\cup Y)}{sup(X)+sup(Y)-sup(X\cup Y)}=\frac{P\left(X\cap Y\right)}{P\left(X\cup Y\right)}$ | The ratio of the probability of X and Y occurring together and X and/or Y occurring together. |
| J-Measure | $P\left(X\cap Y\right)\log\left(\frac{P\left(Y\vert X\right)}{P\left(Y\right)}\right)+P\left(X\cap\bar{Y}\right)\log\left(\frac{P\left(\bar{Y}\vert X\right)}{P\left(\bar{Y}\right)}\right)$ | A measure of cross-entropy. |
| Kappa | $=\frac{P\left(X\cap Y\right)+P\left(\bar{X}\cap\bar{Y}\right)-P\left(X\right)P\left(Y\right)-P\left(\bar{X}\right)P\left(\bar{Y}\right)}{1-P\left(X\right)P\left(Y\right)-P\left(\bar{X}\right)P\left(\bar{Y}\right)}$ | A measure of the agreement between X and Y, larger if X and Y always occur together. |
| Klosgen | $\begin{matrix} \sqrt{sup\left(X\cup Y\right)}\left(conf\left(X\to Y\right)-sup\left(Y\right)\right) \\ =\sqrt{P\left(X\cap Y\right)}\left(P\left(Y\vert X\right)-P\left(Y\right)\right) \end{matrix}$ | Used by the Explora Knowledge Discovery System. Ranges from -1 to 1 with 0 indicating independence. |
| Kulczynski | $\begin{matrix} \frac{1}{2}\left(conf\left(X\to Y\right)+conf\left(Y\to X\right)\right) \\ =\frac{1}{2}\left(P\left(Y\vert X\right)+P\left(X\vert Y\right)\right) \end{matrix}$ | The average confidence of the rule X → Y and the rule Y → X. |
| Goodman-Kruskal Lambda | $\frac{\sum_{x\in X} \left[\max_{y\in Y}P\left(x\cap y\right)-\max_{y\in Y}P\left(y\right)\right]}{n-\max_{y\in Y}P\left(y\right)}$ | The reduction in error for predicting Y when X is known. |
| LaPlace Corrected Confidence | $\frac{c_{XY}+1}{c_{X}+2}$ | A confidence measure that corrects for uncertainty caused by low counts. |
| Least Contradiction | $\frac{sup(X\cup Y)-sup(X\cup\bar{Y})}{sup(Y)}=\frac{P\left(X\cap Y\right)-P\left(X\cap\bar{Y}\right)}{P\left(Y\right)}$ | The difference in support for the rule X → Y and the rule X → not Y, compared to the support for Y. |
| Lerman Similarity | $\sqrt{N}\frac{sup\left(X\cup Y\right)-sup\left(X\right)sup\left(Y\right)}{\sqrt{sup\left(X\right)sup\left(Y\right)}}=\frac{c_{XY}-\frac{c_{X}c_{Y}}{n}}{\sqrt{\frac{c_{X}c_{Y}}{n}}}$ | Support for the rule, centered and scaled. |
| Leverage | $sup(X\cup Y)-sup(X)sup(Y)=P(X\cap Y)-P(X)P(Y)$ | The difference between the support of the rule and the support expected if the antecedent and consequent were independent. |
| Lift | $\frac{conf(X\to Y)}{sup(Y)}=\frac{P\left(X\cap Y\right)}{P\left(X\right)P\left(Y\right)}$ | The ratio of the support of a rule (or itemset) to the expected support if the items were statistically independent. |
| Max Confidence | $\begin{matrix} \max\left(conf\left(X\to Y\right),conf\left(Y\to X\right)\right) \\ =\max\left(P\left(Y\vert X\right),P\left(X\vert Y\right)\right) \end{matrix}$ | The maximum confidence between the rule X → Y and the rule Y → X. |
| Mutual Information | $\frac{\sum_{i\in\left\{X,\bar{X}\right\}} \sum_{j\in\left\{Y,\bar{Y}\right\}} P\left(i\cap j\right)\log\frac{P\left(i\cap j\right)}{P\left(i\right)P\left(j\right)}}{\min\left[-\sum_{i\in\left\{X,\bar{X}\right\}} P\left(i\right)\log P\left(i\right),-\sum_{j\in\left\{Y,\bar{Y}\right\}} P\left(j\right)\log P\left(j\right),\right]}$ | The reduction of entropy (information gain) for Y when X is known. |
| Odds Ratio | $\frac{c_{XY}c_{\bar{X}\bar{Y}}}{c_{X\bar{Y}}c_{\bar{X}Y}}=\frac{P\left(X\cap Y\right)P\left(\bar{X}\cap\bar{Y}\right)}{P\left(X\cap\bar{Y}\right)P\left(\bar{X}\cap Y\right)}$ | The odds of Y occurring if X occurs compared to the odds of Y occurring if X does not occur. |
| Phi Correlation Coefficient | $\frac{nc_{XY}-c_{X}-c_{Y}}{\sqrt{c_{X}c_{Y}c_{\bar{X}}c_{\bar{Y}}}}$ | The Pearson’s product moment correlation coefficient from a contingency table of the antecedent and consequent. |
| Ralambrodrainy Measure | $P\left(X\cap\bar{Y}\right)$ | The support of X → not Y. |
| Relative Linkage Disequilibrium | Based on$D=c_{XY}c_{\bar{X}\bar{Y}}-c_{X\bar{Y}}c_{\bar{X}Y}$ | Compares the support for the rule X → Y to the support expected if X and Y are independent. |
| Rule Power Factor | $sup\left(X\cup Y\right)*conf\left(X\to Y\right)=\frac{P\left(X\cap Y\right)^{2}}{P\left(X\right)}$ | The confidence of a rule weighted by its support. |
| Sebag Measure | $\frac{sup\left(X\cup Y\right)}{sup\left(X\cup\bar{Y}\right)}=\frac{P\left(X\cap Y\right)}{P\left(X\cap\bar{Y}\right)}$ | The support of the rule X → Y compared to the support of the rule X → not Y. |
| Support | $P\left(X\right)$ | The frequency of a rule or itemset. |
| Varying Rates Liaison | $\frac{sup\left(X\cup Y\right)}{sup\left(X\right)sup\left(Y\right)}-1=\frac{P\left(X\cap Y\right)}{P\left(X\right)P\left(Y\right)}$ | Lift shifted to have a neutral value of 0 instead of 1. |
| Yule’s Q | $\frac{OR-1}{OR+1}$ | Normalized odds ratio (OR) to range from -1 to 1. |
| Yule’s Y | $\frac{\sqrt{OR}-1}{\sqrt{OR}+1}$ | Normalized odds ratio (OR) to range from -1 to 1. |

Table S2: Frequency of rule quality measures appearing in the first four principal components identified from each set of rules. Principal component analysis was performed with the R package ‘prcomp’ on the 18 sets on rules (one set for each year-source dataset). The five quality measures with the greatest loadings in each principal component were identified and tabulated across the datasets. The average loading is noted for the two most frequent quality measures in each principal component (bolded).

|  | *Principal Component (Loading)* | | | |
| --- | --- | --- | --- | --- |
| *Quality Measure* | *1* | *2* | *3* | *4* |
| Added Value | 0 | 0 | 0 | 0 |
| Chi-Squared | 3 | 0 | 0 | 0 |
| Certainty Factor | 0 | 10 | 0 | 0 |
| Collective Strength | 0 | 0 | 0 | 1 |
| **Confidence** | 0 | 18 (0.25) | 0 | 0 |
| Conviction | NA | NA | NA | NA |
| Cosine | 16 | 0 | 0 | 0 |
| Count | 0 | 0 | 14 | 0 |
| **Coverage** | 0 | 1 | 0 | 18 (0.37) |
| **Descriptive Confirmed Confidence** | 0 | 18 (0.25) | 0 | 0 |
| Causal Confidence | 0 | 0 | 1 | 16 |
| **Causal Support** | 0 | 0 | 17 (0.37) | 0 |
| Counter-Example Rate | 0 | 0 | 0 | 11 |
| Descriptive Confirm | 0 | 0 | 1 | 17 |
| Difference of Confidence | 1 | 0 | 0 | 0 |
| Fisher’s Exact Test | 0 | 0 | 0 | 4 |
| Gini Index | 3 | 0 | 0 | 0 |
| Hyper Lift | 0 | 0 | 15 | 0 |
| Hyper Confidence | 0 | 0 | 0 | 3 |
| Imbalance Ratio | 0 | 13 | 0 | 0 |
| **Implication Index** | 17 (0.25) | 0 | 0 | 0 |
| Importance | NA | NA | NA | NA |
| Improvement | NA | NA | NA | NA |
| Jaccard Coefficient | 0 | 1 | 0 | 0 |
| J-Measure | NA | NA | NA | NA |
| Kappa |  |  |  |  |
| Klosgen | NA |  |  |  |
| Kulczynski | 6 | 0 | 0 | 0 |
| Goodman-Kruskal Lambda | 13 | 0 | 0 | 0 |
| LaPlace Corrected Confidence | 0 | 16 | 0 | 0 |
| Least Contradiction | 0 | 8 | 2 | 0 |
| Lerman Similarity | 13 | 0 | 0 | 0 |
| Leverage | 0 | 0 | 1 | 0 |
| **Lift** | 0 | 0 | 17 (0.35) | 0 |
| Max Confidence | 0 | 5 | 0 | 2 |
| Mutual Information | NA | NA | NA | NA |
| Odds Ratio | NA | NA | NA | NA |
| **Phi Correlation Coefficient** | 18 (0.24) | 0 | 0 | 0 |
| **Ralambrodrainy Measure** | 0 | 0 | 0 | 18 (0.42) |
| Relative Linkage Disequilibrium | 0 | 0 | 0 | 0 |
| Rule Power Factor | 0 | 0 | 6 | 0 |
| Sebag Measure | NA | NA | NA | NA |
| Support | 0 | 0 | 15 | 1 |
| Varying Rates Liaison | NA | NA | NA | NA |
| Yule’s Q | NA | NA | NA | NA |
| Yule’s Y | NA | NA | NA | NA |
| Average Variance Explained | 0.39 | 0.28 | 0.11 | 0.07 |

Supplementary Figure 1: Average rule quality difference. The average difference in support (green) and confidence (orange) of rules shared between slaughter and retail isolates (A) and between consecutive years (B) for slaughter and retail isolates separately.


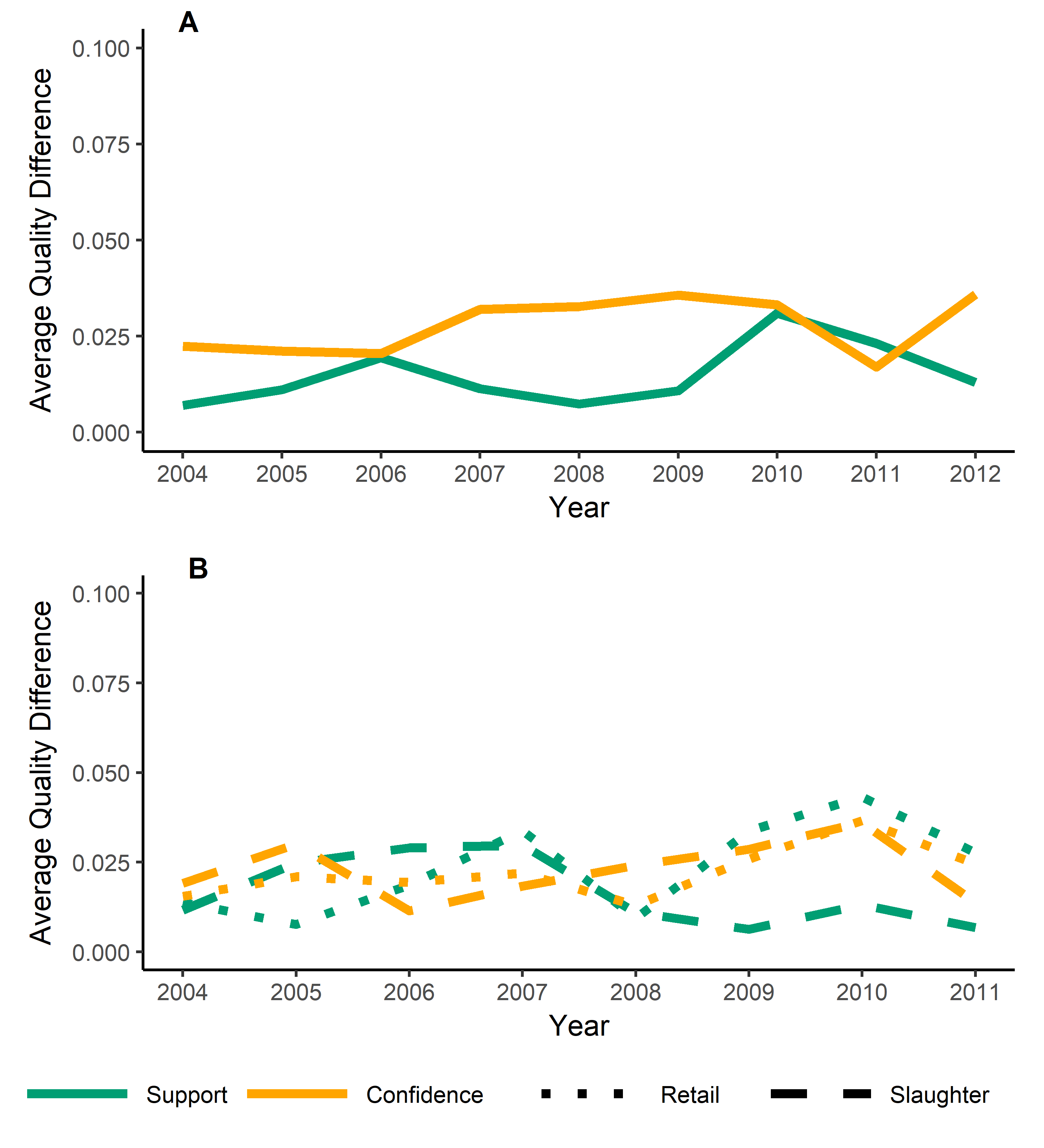


Supplementary Figure 2: Decomposed rule graph density and modularity. Rules were decomposed into nodes (antimicrobials) and edges (connecting antecedent to consequent) and redundant edges were removed. Graph density (A) is the number of edges present divided by the number of possible edges with 15 nodes and is calculated for all possible edges, within-class edges, and between-class edges. Modularity was calculated with edges unweighted (B) or weighted (C) by the number of rules connecting each pair of antimicrobials.


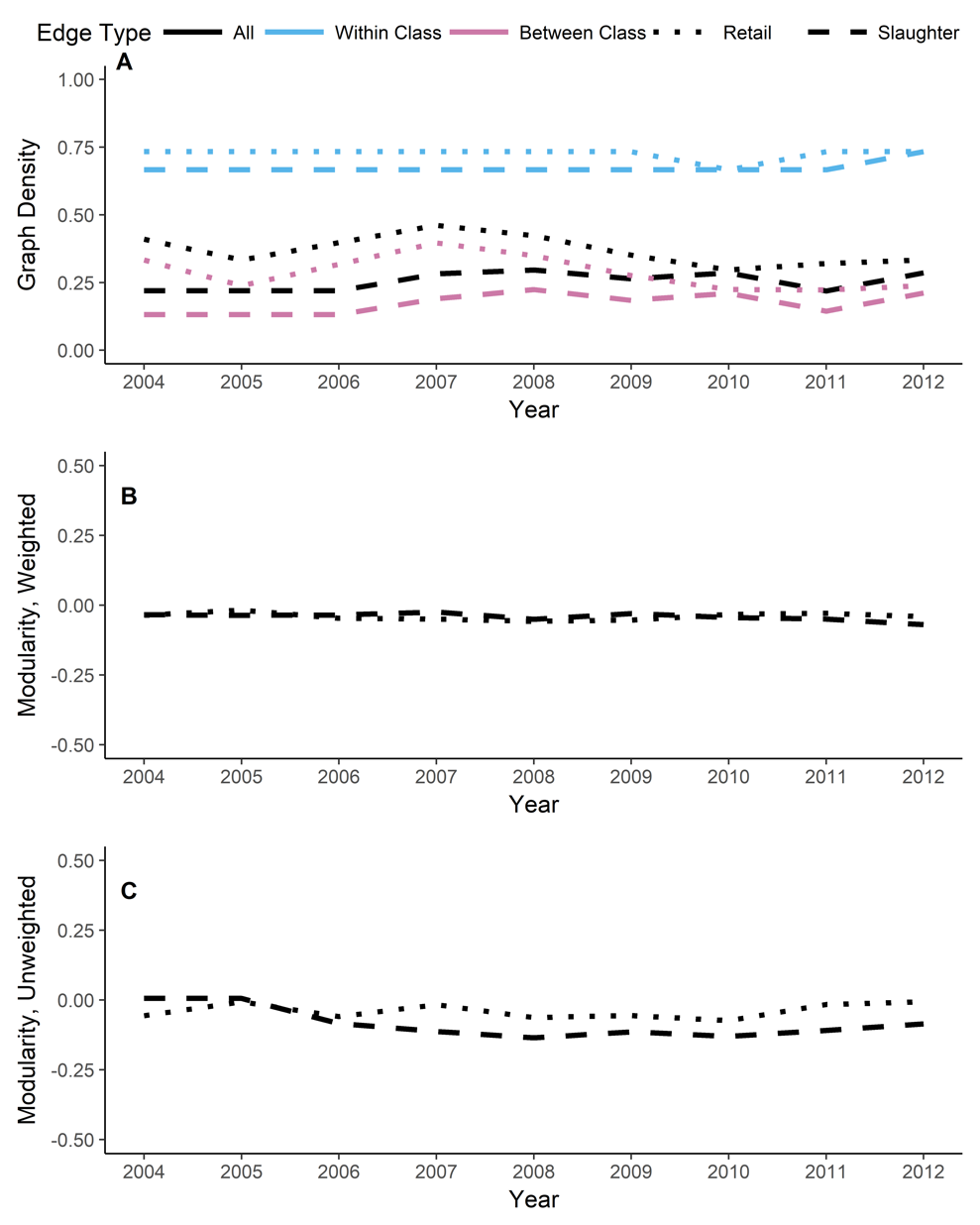


Supplementary Figure 3: Rule overlap between retail datasets and slaughter datasets with equal numbers of isolates within each year (A) and across all years (B). Rule overlap is the proportion of rules shared between two datasets (the number of rules that are in both datasets divided by the total number of rules within the datasets). (A) A slaughter dataset with the same number of isolates as the retail dataset from the same year was created by randomly selecting isolates from all the slaughter isolates in that year. The dataset size each year is given in Table 2, Retail N. (B) Slaughter and retail datasets with equal numbers of isolates within each year and across all years were created by randomly selecting isolates from all slaughter isolates and all retail isolates, respectively. The dataset size is 299 slaughter isolates and 299 retail isolates in each year.


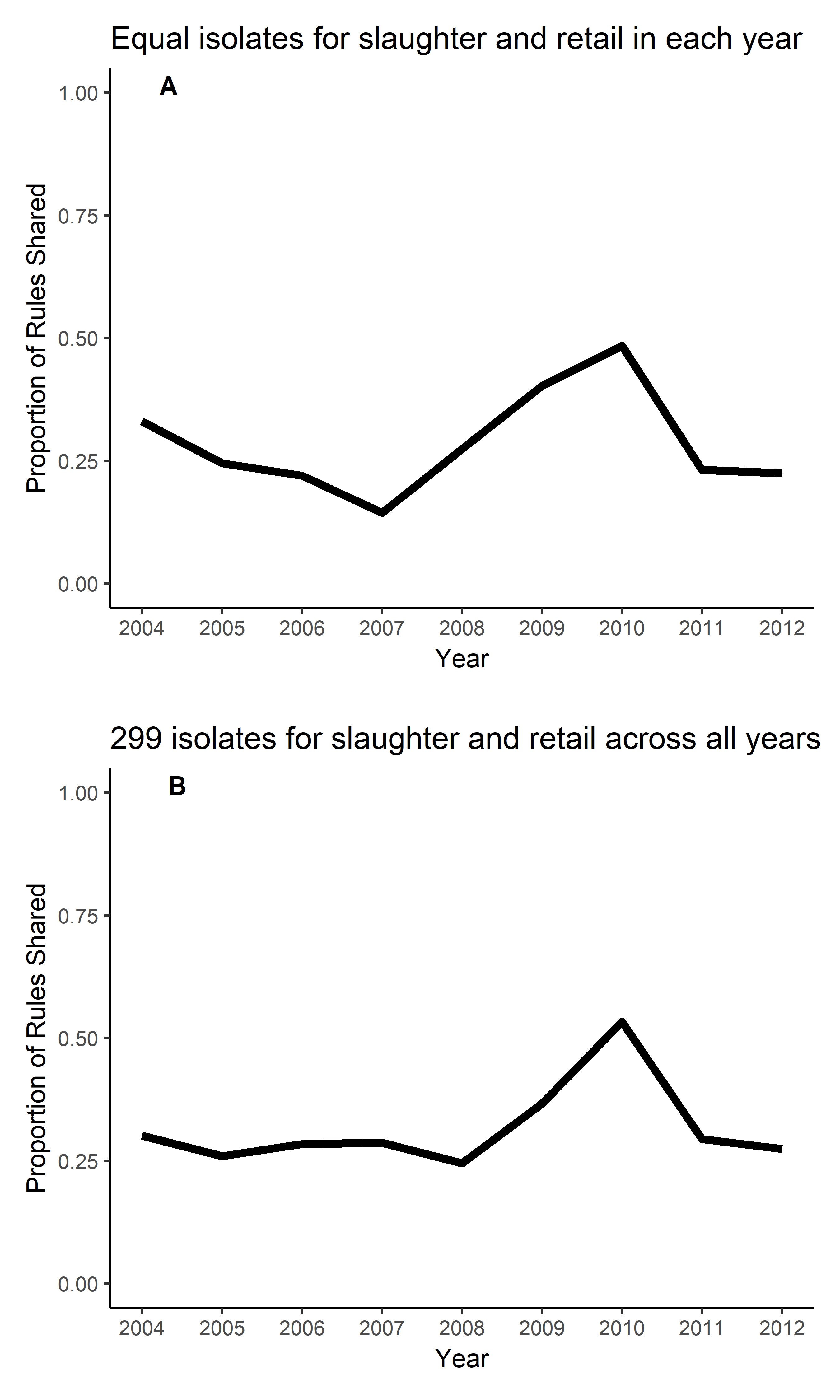

Supplement: Supplementary file 1 [file Table_1.docx]
